# Stromal Remodeling of the Cytokine Secretome Reveals a Chemokine Core Across Breast Cancer and Glioma 3D Cell Culture Models

**DOI:** 10.3390/cells15191728

**Published:** 2026-09-22

**Authors:** David Sergeevichev, Anastasia Leonteva, Alina Kazakova, Maria Bogachek, Anna Ilyina, Vladimir Richter, Anna Nushtaeva

**Affiliations:** 1 Scientific Center of Genetics and Life Sciences, Sirius University of Science and Technology, 1 Olimpiysky Avenue, Sirius (Federal Territory of Sirius), Sochi 354340, Russia; sergeevichev.ds@talantiuspeh.ru (D.S.); bogachek.mv@talantiuspeh.ru (M.B.); ilina.ana@talantiuspeh.ru (A.I.); 2 Meshalkin National Medical Research Center, 15, Rechkunovskaya St., Novosibirsk 630090, Russia; 3 Laboratory of Biotechnology, Institute of Chemical Biology and Fundamental Medicine, Siberian Branch of Russian Academy of Sciences, 8, Akad. Lavrentiev Ave., Novosibirsk 630090, Russia

**Keywords:** tumor microenvironment, cytokines, 3D spheroids, breast cancer, glioma, cancer-associated fibroblasts, endothelial cells, microglia, astrocytes, bootstrap analysis

## Abstract

**Highlights:**

**What are the main findings?**

- The addition of a stromal component to 3D heterotypic cultures was predominantly associated with increased, rather than decreased, cytokine secretion. Out of 256 comparisons, induction was registered in 48.4% of cases, whereas decreased secretion occurred in only 4.3% (an ~11:1 ratio), with the maximal change observed for LIF (log2FC = 8.69).
- Despite tissue-specific differences in the cytokine profiles of breast cancer and glioma cell culture models, a shared set of 14 cytokines dominated by chemokines was identified (7 chemokines, 5 growth factors, and 2 immunoregulatory cytokines).

**What are the implications of the main findings?**

- The observed changes in cytokine secretion depended on both stromal cell type and tumor subtype: CAFs were associated with a broader set of cytokines in comparison to normal fibroblasts. In contrast, triple-negative breast cancer (MDA-MB-231) showed minimal changes, concurrently suppressing GM-CSF and IP-10. This finding underscores the presence of cell- and subtype-specific axes that are susceptible to therapeutic intervention.
- The shared chemokine set identified across the models tested (MCP-1, RANTES, SDF-1α, IP-10, MIP-1α/b, and others) may point to a common axis of stromal–tumor communication and represents a candidate target for further investigation.

**Abstract:**

Mounting evidence indicates that the tumor microenvironment plays a decisive role in cancer progression. However, the quantitative assessment of how individual stromal components contribute to cytokine secretion in 3D models remains poorly understood, especially when comparing tumours of different origins. The development of 16 groups of 3D spheroid cultures, encompassing stromal monocultures, two-component models (tumor + endothelium/glia), and three-component models (tumor + endothelium + fibroblast; glioma + astrocytes + microglia), was conducted. These models represent breast cancer and glioma cell culture models. The secretion of 32 distinct cytokines was measured using xMAP technology. Induction was defined as log2FC ≥ 1 with bootstrap-estimated 95% confidence intervals. The induced cytokines were subsequently classified into two operational categories: “emergent” (stromal monoculture level below 3D-2) or “stroma-contributing” (stromal level ≥ 3D-2). In the breast cancer model, a consensus across all cell lines identified seven cytokines (HGF, IL-10, LIF, MCP-1, MCP-3, MIP-1α, and SCGF-β). Cancer-associated fibroblasts (CAFs) were associated with a broader spectrum than normal fibroblasts (28 vs. 23), exhibiting a complete overlap in their repertoire. Triple-negative breast cancer (MDA-MB-231) showed minimal secretion (7–8 cytokines), with decreased secretion of GM-CSF and IP-10; b-FGF levels were reduced in six of eight comparisons. In the glioma model based on U87MG cells, the addition of astrocytes (CCF-STTG1) coincided with increased secretion of 13 different cytokines, whereas microglia (HMC3) were associated with 8 cytokines. The cross-tumor intersection comprised 14 cytokines (7 chemokines, 5 growth factors, and 2 immunoregulatory cytokines), with maximal production observed for LIF, IL-8, MCP-3, MIP-1α, and IL-6. These findings indicate that the addition of stromal cells is associated with both common and tissue-specific changes in the cytokine profile, and that CAFs display a broader secretory repertoire than normal fibroblasts in this model.

## 1. Introduction

Cancer is the second leading cause of death after cardiovascular disease and remains a serious public health problem both in the Russian Federation and worldwide [1]. Breast cancer (BC) is the most prevalent form of malignant neoplasm in women, with brain metastases being a primary cause of mortality [2]. In contrast, gliomas, which are primary brain tumors, are known for their profound resistance to therapy and unfavorable prognoses [3].

Cytokines are a class of small protein or polypeptide molecules that are secreted by immune, tumor, and stromal cells. By binding to receptors on the surface of target cells, they trigger signaling cascades that regulate growth, differentiation, and the immune response. The process of cytokine communication is facilitated by autocrine, paracrine, and endocrine mechanisms [4].

The tumor microenvironment (TME) is a dynamic and heterogeneous ecosystem in which malignant cells directly or indirectly interact with stromal, immune, endothelial cells, and extracellular matrix (ECM) proteins [5]. The TME, through a complex network of intercellular communications and molecular signals, including cytokines, actively participates in tumor progression, immune evasion, and the development of therapy resistance [6]. Cytokine imbalance within the TME has been identified as a pivotal factor in the maintenance of chronic inflammation, angiogenesis, and immunosuppression, thereby fostering tumor growth [7]. Pro-inflammatory cytokines, such as IL-2, IL-12, IL-15, and type I/II interferons, have been shown to activate cytotoxic T lymphocytes and NK cells, thereby stimulating their ability to destroy tumor cells [8]. In contrast, immunosuppressive cytokines, including IL-10, IL-4, IL-6, and TGF-β, have been shown to suppress effector lymphocytes and promote the recruitment and expansion of regulatory T cells, myeloid-derived suppressor cells, and tumor-associated macrophages, thereby weakening antitumor immunity [9]. A more profound comprehension of the mechanisms underlying the modulation of tumor cell behavior and the TME by cytokines may identify novel therapeutic targets [10]. It is imperative to underscore the fact that both breast and brain tumors employ inflammation and immunosuppression to facilitate their growth. However, it is equally crucial to acknowledge that their microenvironments exhibit substantial disparities, contingent on factors such as tissue location and molecular subtypes.

To overcome the limitations of monolayer cultures and in vivo models, systems that recapitulate tumor architecture and the complexity of its microenvironment are needed [11]. Among the promising in vitro platforms, heterotypic spheroids composed of tumor cells and cells from the microenvironment more accurately mimic the tissue architecture, diffusion limitations, and intercellular interactions characteristic of solid tumors [12,13]. Spheroids are formed by the self-assembly of cells into multicellular aggregates that generate physiological gradients of oxygen, nutrients, and metabolites. These gradients are absent in monolayer cultures [14]. A distinguishing feature of these models is their capacity for secretory activity, particularly the production of cytokines that regulate inflammation, the immune response, angiogenesis, and tissue homeostasis. A fundamental question that determines the validation of these models and the interpretation of the resulting data is the extent to which the secretory patterns of heterotypic spheroids are universal—that is, common to different cell combinations—or, conversely, specific to a particular cell composition.

A comprehensive analysis of the existing dataset reveals a substantial correlation between the secretory profiles of heterotypic spheroids and the tumor type, as well as the composition of the cellular microenvironment. For instance, in a heterotypic spheroid model of breast cancer, which incorporates CAFs, an aggressive and immunosuppressive phenotype was identified, as well as a distinct cytokine cluster (LIF, SDF-1α, HGF, SCGF-β), which may play a role in regulating PD-L1 expression by tumor cells [13]. An immunosuppressive cytokine profile was identified, characterized by increased concentrations of pro-inflammatory chemokines, such as CCL2, CCL10, and IL-6, while concurrently decreasing the levels of T-cell chemoattractants, including CCL4, CCL5, and CCL9. This profile was detected in heterotypic pancreatic adenocarcinoma spheroids [15]. The co-cultivation of glioblastoma cells and microglia has been demonstrated to result in the production of chemoattractants, including CSF-1, CXCL12 (SDF-1α), and GM-CSF. These factors have been shown to stimulate microglia migration to the tumor mass and the formation of a pro-tumorigenic niche [16].

The molecular subtypes of breast cancer (BC) exhibit substantial disparities in the composition and activity of the TME. The basal-like and HER2-positive subtypes are distinguished by heightened immune infiltration—a phenomenon referred to as “immune hot”—in contrast to the “immune cold” state observed in luminal subtypes [17]. Triple-negative BC (TNBC) has been shown to exhibit a pronounced mesenchymal phenotype, characterized by autonomous production of pro-inflammatory cytokines, which can reduce its dependence on stromal stimulation. In general, the interaction with fibroblasts differs between subtypes in terms of the spectrum and the amplitude of induced cytokines [18]. In the glioma model, astrocytes and microglia exhibit distinct patterns of paracrine signaling. Specifically, astrocytes predominantly induce emergent secretion via the JAK/STAT pathway, while microglia contribute directly to secretion [18,19]. Nevertheless, the quantitative comparison of stromal-induced cytokine programs in tumors of different histogenesis within a single experimental and statistical paradigm remains a poorly understood task.

In a previous study, an identical BC model system was utilized to identify the LIF/SDF-1α/HGF/SCGF-β cytokine cluster as a potential regulator of PD-L1 expression [13]. The present study extends this approach in three directions. Firstly, a heterotypic 3D glioma model was incorporated into the analysis, thereby enabling cross-tumor comparison. Secondly, a single, stringent statistical criterion (log2FC bootstrap estimator with a 95% confidence interval) was applied to objectively identify induced cytokines. Thirdly, a threshold-independent annotation of the secretion pattern was performed for each induced cytokine, distinguishing between “stroma-contributing” and “emergent” states.

The aim of the present study was to identify cytokines whose secretion is reliably induced by the addition of stromal cells in 3D spheroid models of BC and glioma cell lines. In addition, the study sought to determine the cross-tumor intersection of these cytokines and to characterize the secretion patterns.

## 2. Materials and Methods

### 2.1. Nomenclature of Heterotypic 3D Cultures and Comparison Design

To systematically analyze the impact of the stromal microenvironment on the secretory profile of tumor cells, a pairwise comparison design was employed, in which each three-component co-culture was compared against its corresponding two-component culture. All combinations were encoded according to a nomenclature in which each cell line was assigned a unique letter code, and the digit “n” in the 3D-n designation reflects the total number of cell lines within the construct, irrespective of their type. This ensures unambiguous identification of the composition of each experimental group (Table 1).

For breast cancer, six comparisons were performed, corresponding to the addition of either normal (BN120f) or cancer-associated (BrC4f) fibroblasts to 3D-2 cultures comprising each of the three tumor cell lines co-cultured with TIME endothelium cell. For glioma, two comparisons were performed, in which the missing stromal component, microglia (HMC3) or astrocytes (CCF-STTG1), was added to the 3D-2GA or 3D-2GM cultures, respectively.

### 2.2. Cell Lines

The study employed breast cancer cells of three molecular subtypes: SK-BR-3 (HER2+, ER-/PR-) (ATCC, #HTB-30, Manassas, VA, USA); MCF7 (luminal A, ER+/PR+/HER2-) (#ACC 115, DSMZ, Braunschweig, Germany); and MDA-MB-231 (triple-negative, ER-/PR-/HER2-) (#ACC 65, DSMZ, Braunschweig, Germany). U87MG glioma cells—purchased from the American Type Culture Collection (ATCC), Manassas, VA, USA—were also used. As the endothelial component for breast cancer models, TIME (Telomerase Immortalized Microvascular Endothelial) cells were used, kindly provided by Dr. I. Vakhrushev (V.N. Orekhovich Research Institute of Biomedical Chemistry, Moscow) and Dr. Pavel Makarevich (Centre for Regenerative Medicine, Lomonosov Moscow State University, Moscow). BrC4f and BN120f were obtained in the Laboratory of Biotechnology in the Institute of Chemical Biology and Fundamental Medicine SB RAS (ICBFM, Novosibirsk, Russia). The stromal cells included normal breast fibroblasts BN120f (Biotechnology Laboratory Collection, ICBFM SB RAS, Novosibirsk), derived from healthy breast tissue [20,21]. From the same collection, the BrC4f fibroblast culture was used, which was obtained from a tumor sample of a patient with a confirmed breast cancer diagnosis. This cell line was previously characterized as cancer-associated fibroblasts (CAFs) [21,22]. For U87MG-based glioma models (ATCC #HTB-14, Manassas, VA, USA), HMC3 microglial cells (#C0005019, AddexBio, San Diego, CA, USA) and CCF-STTG1 astrocyte-like cells (#C0005058, AddexBio, San Diego, CA, USA)—purchased from SIAB, Moscow, Russia—served as stromal components of the tumor microenvironment. The absence of mycoplasma contamination in all cell lines was confirmed through RT-PCR analysis (Biolabmix, Novosibirsk, Russia). The genetic identification of cell lines was performed using the GOrDIS Plus kit (GORDIZ, Moscow, Russia). The STR profiles correspond to those published in international databases, namely ATCC, DSMZ, and Cellosaurus.

Stromal fibroblasts BrC4f and BN120f and MCF7 immortalized tumor cells were cultured in IMDM medium (#I7633-10X1L, Sigma-Aldrich, St. Louis, MO, USA). MDA-MB-231, SK-BR-3, CCF-STTG1, and HMC3 cells were cultured in DMEM/F12 medium (#42400028, Thermo Fisher/Gibco, New York, NY, USA). U87MG cells were cultured in αMEM medium (#3075927, Thermo Fisher/Gibco, Paisley, UK), and TIME microvascular endothelial cells were cultured in EndoLife medium (Appscience, Moscow, Russia). All culture media were supplemented with 10% fetal bovine serum (#A316040, Thermo Fisher, Waltham, MA, USA), 1× GlutaMAX™ solution (#35050061, Thermo Fisher/Gibco, New York, NY, USA), and 1× antibiotic-antimycotic solution (#15140122, Thermo Fisher/Gibco, Waltham, MA, USA). In addition to the above components, the medium for SK-BR-3 cells also contained 1× MEM non-essential amino acids (#11140050, Thermo Fisher/Gibco, Waltham, MA, USA) and 1× sodium pyruvate (#11360070, Thermo Fisher/Gibco, Waltham, MA, USA). All cell lines were cultured in tissue culture-treated flasks (TPP, Trasadingen, Switzerland). Upon reaching confluence, cells were detached using TrypLE™ (#12604013, Invitrogen, Waltham, MA, USA), diluted with complete cell-specific growth medium at a ratio of 1:3–1:4 (*v*/*v*), and subcultured. Cell count and viability were determined using a LUNA-II™ automated cell counter (Logos Biosystems, Anyang-Si, Republic of Korea). All cultures were maintained at 37 °C in a humidified atmosphere of 5.0 ± 0.5% CO_2_ (standard conditions).

The study protocol (Report #1, 14 March 2017) was approved by the Institute of Molecular Biology and Biophysics SB RAMS Ethics Committee in accordance with the Declaration of Helsinki of 1975. All patients provided written informed consent.

### 2.3. Heterotypic Spheroid Models

In 3D-2 breast cancer models, a cell ratio of 1:2 (tumor:endothelial) was maintained; in 3D-3 models, the ratio was 1:4:2 (tumor:stromal:endothelial). Thus, the proportion of tumor cells was the lowest across all breast cancer constructs. For glioma models, the ratio was 3:2 in 3D-2 models (glioma:cells of the tumor microenvironment) and 3:2:2 (tumor:astrocytes:microglia) in the 3D-3GAM model. Previous studies have described the morphofunctional characteristics of heterotypic spheroid models, including glioma and breast cancer, and their interaction with immune cells such as NK cells [12,13,23]. The order of proportions corresponds to the order of cell lines listed for each model in Table 1.

Cells were cultured in 24-well Nunclon™ Sphera™ plates (#174930, Thermo Fisher, MA, USA) at a density of 500,000 cells per well under standard conditions in serum-free DMEM/F12 medium (Thermo Fisher/Gibco, USA) supplemented with 20 ng/mL epidermal growth factor (#E9644, Sigma-Aldrich, Burlington, MA, USA), 20 ng/mL basic fibroblast growth factor (#PHG0261, Thermo Fisher/Gibco, New York, NY, USA), 5 µg/mL insulin (#I9278, Sigma-Aldrich, Burlington, MA, USA), 2% B-27™ Plus Supplement (#A35828010, Thermo Fisher/Gibco, Burlington, MA, USA), 1% N-2 Supplement (#17502048, Thermo Fisher/Gibco, Grand Island, NY, USA) and 0.4% BSA (#126593, Sigma-Aldrich, Burlington, MA, USA). The growth factor cocktail was adapted from a previously published protocol [24]. An inverted Nexcope NIB620 microscope (Novel Optics, Ningbo, China) was used to monitor spheroid formation.

### 2.4. Collection of Biological Samples

The procedure for the formation of spheroids is to be conducted in line with the established protocol, as set out in Section 2.3 [12,13,23]. After 5 days of spheroid cultivation, the spheroids from each well were transferred to centrifuge tubes. Subsequently, the samples were subjected to a centrifugal process at a speed of 1000× *g* for a duration of 15 min at a temperature of 4 °C. The supernatant was used for further analyses, while the cell pellet was used for total protein estimation. All models were assembled in parallel from an equal starting cell number (500,000 cells per spheroid) in an equal volume of medium. The culture supernatant was collected and filtered through a 0.22 µm filter (TPP Techno Plastic Products AG, Trasadingen, Switzerland). BSA was added to a final concentration of 0.5%, in accordance with the manufacturer’s recommendations for the Bio-Plex Pro Human Cytokine Screening 48-Plex Panel (#12007283, Bio-Rad Laboratories, Hercules, CA, USA), after which it was stored in aliquots at −80 °C. The spheroid pellet was washed twice with 1x PBS and then subjected to centrifugation at 1000× *g* for five minutes at 4 °C, after which it was collected for subsequent analysis of total protein.

### 2.5. Total Protein Assay

The spheroid pellet was lysed in RIPA buffer containing a 1× protease inhibitor cocktail (Servicebio, Wuhan, China) at the following concentrations: 150 mM NaCl, 1% Triton X-100, 0.1% SDS, and 50 mM Tris-HCl (pH 7.4). The protein content of the lysates was determined using a Bradford-based protein assay kit (FineTest, Wuhan, China). Contamination by extracellular proteins was monitored further by collecting and analysing the supernatant from the final wash in parallel. No protein signal was detected in these samples. To account for differences in final biomass between the compared models, total protein content was measured in whole-cell lysates from each culture condition at the end of the experiment. A normalization coefficient (factor) was calculated for each model as the ratio of its total protein content to the lowest total protein value observed across all models: *k_i_ = P_i_*/*P_min_*, where *P_i_* is the total protein content of model *i*, and *P_min_* is the minimum total protein value across all models. Cytokine concentrations were then divided by the corresponding coefficient *k_i_* to obtain biomass-adjusted values (see Table S2).

Any differences in final biomass should be interpreted as an integrated outcome reflecting both intercellular interactions and variations in proliferation, survival, and/or cell composition. All experiments were performed in three biological replicates (*n* = 3).

### 2.6. Cytokine Secretion Assay

Concentrations of cytokines, chemokines, and growth factors secreted by 3D models into the culture medium were measured using xMAP technology with the Bio-Plex Pro Human Cytokine Screening 48-Plex Panel according to the manufacturer’s protocol (#12007283, Bio-Rad Laboratories, Hercules, CA, USA). Briefly, the previously obtained supernatant was incubated with antibody-coupled magnetic beads for 16 h at 4 °C. After washing, detection antibodies were added and incubated for 30 min, followed by incubation with streptavidin-phycoerythrin for 10 min. All incubation steps were performed under continuous shaking at 300 rpm. Lyophilized standards were reconstituted in standard diluent, and serial dilutions were performed to generate a standard curve.

Measurements were performed on a QuattroPlex analyzer (Dia-M, Moscow, Russia) according to the manufacturer’s instructions. For each analyte, median fluorescence intensity (MFI) was recorded, and concentrations were calculated from standard curves using QuattroPlex Lab software (version 1.5.3). For analytes with concentrations below the lower limit of quantification (LLOQ), a value of LLOQ/2 was assigned for statistical analysis. An additional control was employed: the culture medium used to cultivate the spheroid models was used, and the values obtained for this control were subtracted from those of the experimental samples. The data were analysed as absolute concentrations in pg/mL, normalised to total protein content. Normalisation was performed by dividing the absolute cytokine concentration by the normalisation factor (see Table S2). All experiments were performed in three biological replicates (*n* = 3).

### 2.7. Effect Size Estimation

To assess changes in cytokine secretion, an effect-size approach with bootstrap estimation of confidence intervals was employed. For each comparison and each cytokine, the log2 fold change (log2FC) was calculated as: log2FC = log2 (mean concentration in 3D-3/mean concentration in 3D-2). Positive log2FC values therefore indicate increased secretion in the 3D-3 cultures, and negative values indicate decreased secretion. A small constant (1 × 10^−6^) was added to both numerator and denominator to avoid division by zero. To estimate the confidence interval (CI), bootstrap resampling (*n* = 1000 iterations) was performed by sampling the original values with replacement. The median and 95% CI (2.5th and 97.5th percentiles) were determined from the distribution of bootstrap log2FC estimates. The reported log2FC values correspond to the median of this bootstrap distribution.

A cytokine was classified as “induced” when both of the following conditions were met: log2FC ≥ 1 (two-fold or greater increase) and the lower bound of the 95% CI exceeded 0. A cytokine was classified as “suppressed” when log2FC ≤ −1 and the upper bound of the 95% CI fell below 0. In all other cases, the cytokine was assigned to the “unchanged” category. No correction for multiple comparisons was applied. These designations simply mirror the statistical shift in changes, without suggesting a particular mechanism or cellular basis. The results were interpreted as exploratory, with consensus across cell lines regarding cytokine secretion serving as an additional biological filter for false positives.

### 2.8. Annotation of the Secretion Pattern: “Emergent” vs. “Stroma-Contributing”

For each induced cytokine, a threshold-independent annotation of the likely secretion pattern was performed. The mean cytokine concentration of the stromal component (3D-1N, 3D-1C, 3D-1A, or 3D-1M) was compared with the mean concentration in the 3D-2 breast cancer or glioma culture. If the stromal component secreted the cytokine at a level lower than that in the 3D-2 culture, the cytokine was annotated as “emergent” (i.e., increased secretion in the 3D-3 culture was not detected in the corresponding stromal monoculture). If the stromal component secreted the cytokine at a level equal to or greater than that in the 3D-2 culture, the cytokine was annotated as “stroma-contributing” (i.e., the increase in cytokine level in the 3D-3 culture may involve an additive stromal contribution).

### 2.9. Functional Classification of Cytokines

For the purposes of differential analysis and visualization, cytokines were divided into three functional groups according to their canonical roles in the tumor microenvironment [24,25,26,27]:

- Chemokines (*n* = 11): CTACK (CCL27), Eotaxin (CCL11), GRO-α (CXCL1), IL-8 (CXCL8), IP-10 (CXCL10), MCP-1 (CCL2), MCP-3 (CCL7), MIP-1α (CCL3), MIP-1β (CCL4), RANTES (CCL5), SDF-1α (CXCL12).
- Growth factors (*n* = 9): b-FGF, G-CSF, GM-CSF, HGF, M-CSF, SCGF-β, VEGF, β-NGF, LIF.
- Immunoregulatory cytokines (*n* = 12): IFN-α2, IFN-γ, IL-1α, IL-1β, IL-1ra, IL-2Rα, IL-6, IL-10, IL-15, TNF-α, TNF-β.

### 2.10. Aggregation of Results and Cross-Tumor Comparison

For breast cancer, results are presented both for each cell line individually and as either a consensus—i.e., cytokines induced across all three tumor cell lines (3/3 consensus) or in at least two lines (2/3 consensus)—or a union (i.e., cytokines induced in at least one tumor cell line). For glioma, results are presented for each comparison separately. The union across the stroma-addition comparison groups (3D-2GA → 3D-3GAM and 3D-2GM → 3D-3GAM) was used for cross-tumor comparison. The cross-tumor intersection was defined as the set of cytokines induced in both breast cancer (union across cell lines and stromal types) and glioma (union across stromal additions).

### 2.11. Multivariate Analysis of Secretion Profiles

To visualize the overall structure of secretion profiles, principal component analysis (PCA) was applied to log-transformed (log2(value + 1)) and standardized (z-score) cytokine concentrations. Samples were displayed in the coordinates of the first two principal components, color-coded by culture complexity and by tumor model (breast cancer, glioma).

### 2.12. Analysis Software

All statistical analyses and visualizations were performed in Python (v.3.12). Data processing and aggregation relied on pandas (v.2.2.2) and NumPy (v.2.0.2) libraries; statistical computations, including bootstrap estimation, used SciPy (v.1.16.3) and scikit-learn (v.1.6.1). All figures were generated using matplotlib (v.3.10.0) and seaborn (v.0.13.2). Bootstrap estimation was performed with a fixed random seed to ensure full reproducibility. The complete analysis pipeline, including Jupyter notebooks documenting all computational steps, is available from the corresponding author upon reasonable request.

## 3. Results

### 3.1. General Characteristics of Secretome Induction

The primary analysis revealed that 32 of the 48 analytes exhibited analytical value: CTACK, Eotaxin, b-FGF, G-CSF, GM-CSF, GRO-a, HGF, IFN-α2, IFN-γ, IL-1α, IL-1β, IL-1ra, IL-2Rα, IL-6, IL-8, IL-10, IL-15, IP-10, LIF, MCP-1, MCP-3, M-CSF, MIF, MIP-1α, MIP-1β, β-NGF, RANTES, SCGF-β, SDF-1α, TNF-α, TNF-β, and VEGF. The secretion of the remaining cytokines did not exceed the background values. Of the 256 comparisons performed (eight 3D-2 → 3D-3 transitions × 32 analytes), 124 cytokines (48.4%) were classified as induced, 11 cytokines (4.3%) as suppressed, and 121 (47.3%) as unchanged. Consequently, the addition of stromal cells into 3D-2 cultures elicited a prominent and predominantly favorable remodeling of the cytokine secretome in both tumor models. Secretion suppression was an infrequent occurrence, manifesting predominantly in a b-FGF manner among the cases examined (six of eleven cases exhibited suppression).

The secretion profiles were analyzed using principal component analysis (PCA), which revealed a separation of the samples according to both culture complexity (3D → 3D-2 → 3D-3) and tumor model (BC vs. glioma) (Figure 1).

The 3D-3 cultures occupied distinct regions of the principal component space, without mixing with either stromal or 3D-2 cultures. This finding is consistent with the pronounced secretome remodeling that occurred with the addition of stromal cells. The distinction between breast cancer and glioma suggests that tumour-specific secretion profiles are maintained, despite characteristics associated with pathogenesis being similar.

### 3.2. Cytokine Induction in a Breast Cancer Model

For breast cancer specifically, three heterotypic tumor models were analyzed. These models were based on the MCF7, SK-BR-3, and MDA-MB-231 lines. The models were analyzed with normal BN120f fibroblasts and cancer-associated BrC4f fibroblasts. The complete map of log2FC changes for all six breast cancer comparisons is presented in Figure 2. The cell lines exhibited substantial variation in their inducer response breadth. The addition of normal fibroblasts or CAFs prompted the secretion of 16 to 27 cytokines in 3D-LEN and 3D-3HEN lines, while only 7–8 cytokines were observed in 3D-3TEN (Figure 2, Table S1).

#### 3.2.1. Induction by Addition of Normal Fibroblasts

Adding BN120f cells to 3D-2 breast cancer cultures increased the secretion of 20, 16, and 7 cytokines in 3D-3HEN, 3D-3LEN, and 3D-3TEN, respectively. The five cytokines whose production was stimulated in all three breast cancer cell lines by the addition of BN120f (i.e., a 3/3 consensus) were: HGF, IL-10, LIF, MCP-3 and SCGF-β. The 2/3 consensus encompassed 15 distinct cytokines. The array of cytokines and chemokines included Eotaxin, G-CSF, GRO-a, HGF, IFN-α2, IL-10, IL-1β, IL-6, IL-8, LIF, MCP-1, MCP-3, MIP-1α, SCGF-β, and TNF-β (Table S1).

The most significant induction with the addition of BN120f was observed for G-CSF (log2FC = 6.34 in 3D-3LEN), IL-8 (6.62 in 3D-3HEN), IL-6 (5.44 in 3D-3HEN), and LIF (5.68 in 3D-3LEN; 4.78 in 3D-3HEN) (Table S3).

#### 3.2.2. Induction by Adding CAF

The addition of BrC4f cells resulted in the secretion of 27 cytokines in 3D-3HEC, 25 cytokines in 3D-3LEC, and 8 cytokines in 3D-3TEC. The 3/3 consensus for BrC4f addition was seven cytokines: HGF, IL-10, LIF, MCP-1, MCP-3, MIP-1α, and SCGF-β. The 2/3 consensus encompassed 25 cytokines (see Table S1). The most significant induction with the addition of BrC4f was observed for LIF (log2FC = 8.69 in 3D-3HEC; 7.72 in 3D-3LEC; 5.47 in 3D-3TEC), MCP-3 (7.16 in 3D-3LEC; 6.61 in 3D-3HEC; 6.17 in 3D-3TEC), IL-8 (7.08 in 3D-3HEC), and MIP-1α (5.61 in 3D-3LEC; 5.39 in 3D-3HEC; 4.87 in 3D-3TEC) (Figure 2, Table S3).

#### 3.2.3. Consensus Across All Breast Cancer Cell Lines and Stroma

When combining both fibroblast types, a 3/3 consensus identified seven cytokines: HGF, IL-10, LIF, MCP-1, MCP-3, MIP-1α, and SCGF-β. The secretion of these cytokines is produced independently of the molecular subtype of breast cancer or the type of fibroblasts added. This represents the core of the stromal-induced breast cancer secretome. The intersections of the sets of secreted cytokines among the three BC cell lineages are illustrated in Figure 3.

A core group of seven consensus 3/3 cytokines was characterized by an increase in all breast cancer cell lines, while the majority of cytokines were induced only in MCF7 and/or SK-BR-3, but not in MDA-MB-231. The pooling of data across all lines and stromata yielded a total of 28 cytokines, out of the 32 included in the study (Table S1).

#### 3.2.4. Comparison of BN120f and BrC4f Fibroblasts

In a study of 3D-2 breast cancer cell cultures, the addition of cancer-associated BrC4f fibroblasts was found to induce the secretion of a broader spectrum of cytokines when compared to the secretion elicited by normal BN120f fibroblasts. Significant differences in the number of cytokines were found in SK-BR-3 (27 versus 20), MCF7 (25 versus 16), and MDA-MB-231 (8 versus 7) cells following analysis. Across all cell lines, BrC4f induced a total of 28 cytokines, while BN120f induced only 23 cytokines, with all BN120f-induced cytokines being part of the BrC4f repertoire. Five cytokines, including GM-CSF, IL-1α, IL-2Rα, MIF and RANTES, were observed exclusively in BrC4f-containing models (Table S1).

At the 2/3 consensus level (detection in at least two cell lines), 10 cytokines were found to be BrC4f-specific: CTACK, GM-CSF, IL-2Rα, IP-10, M-CSF, MIP-1β, RANTES, SDF-1α, TNF-α, and β-NGF. Furthermore, the investigation revealed that cytokines induced exclusively by BN120f (and not BrC4f) were absent at both levels of aggregation. Furthermore, all five cytokines of the 3/3 consensus level determined for BN120f were also induced by BrC4f fibroblasts. This finding suggests that BrC4f exhibits a broader range of inducer activity toward BN120f, resulting in the induction of additional chemokines (MIP-1β, SDF-1α, RANTES), growth factors (GM-CSF, M-CSF), and immunoregulatory cytokines (TNF-α, IL-2Rα) (see Table S1 for details).

#### 3.2.5. Features of the MDA-MB-231 Cells

The triple-negative MDA-MB-231 subtype had the lowest response, with a range of 7–8 cytokines, depending on the type of added fibroblasts. In contrast, MCF7 and SK-BR-3 demonstrated a response characterized by the secretion of 16–25 and 20–27 cytokines, respectively. Furthermore, the MDA-MB-231 cell line exhibited a quantitative inferiority to the other lines examined. All eight cytokines induced in the MDA-MB-231 cell line (HGF, IL-10, LIF, MCP-1, MCP-3, MIP-1α, RANTES, SCGF-β) were also induced in the MCF7 and SK-BR-3 cell lines. Meanwhile, none of the 17 cytokines detected in the other two lines reached the induction threshold in the MDA-MB-231 line (Figure 3). The addition of fibroblasts decreased the secretion of GM-CSF and IP-10 (see Section 3.5, “Suppression of Secretion”). These results indicate that the MDA-MB-231-based models showed a comparatively limited change in cytokine secretion following fibroblast addition, which merits further investigation.

### 3.3. Cytokine Induction in a Glioma Model

In the glioma model based on the U87MG tumor cell line, two comparisons were analyzed. These corresponded to the addition of a third cell type to each of the 3D-2 cultures (Figure 4).

#### 3.3.1. Adding Microglia to the 3D-2GA Model

Adding HMC3 cells to the 3D-2GA model resulted in the secretion of eight cytokines, five of which were chemokines (Figure 4). The highest increment was demonstrated by RANTES (log2FC = 4.72), β-NGF (3.75), and IP-10 (3.19); moderate induction was observed for GRO-α (2.86), MIP-1α (2.83), G-CSF (1.83), GM-CSF (1.53), and MIP-1β (1.45) (Table S3). According to the secretion pattern annotation (Section 2.8), MIP-1α and G-CSF were classified as “emergent”, and the remaining six cytokines were classified as “stroma-contributing”.

#### 3.3.2. Adding Astrocytes to the 3D-2GM Model

Adding CCF-STTG1 astrocytes to 3D-2GM spheroids resulted in the secretion of 13 cytokines (Figure 4). The most significant increases were observed for IP-10 (log2FC = 3.05), SDF-1α (2.99), and VEGF (2.66). Moderate induction was noted for MCP-1 (1.89), MIP-1α (1.87), HGF (1.73), GRO-α (1.70), β-NGF (1.58), RANTES (1.4), G-CSF (1.12), IL-6 (1.07), TNF-α (1.13), and LIF (1.06) (Table S3). According to the secretion pattern annotation, nine of the 13 cytokines were classified as “emergent”, including G-CSF, GRO-α, IL-6, IP-10, LIF, MIP-1α, RANTES, TNF-α, and β-NGF. The remaining four cytokines were classified as “stroma-contributing”, namely HGF, MCP-1, SDF-1α, and VEGF. The prevalence of the “emergent” pattern (69%) may be consistent with paracrine signaling associated with the presence of astrocytes.

#### 3.3.3. Union by Stroma Addition

The combined list of cytokines produced when the stromal component (HMC3 or CCF-STTG1) was added to 3D-2 glioma cultures comprised 15 cytokines, seven of which were chemokines: GRO-α, IP-10, MCP-1, MIP-1α, MIP-1β, RANTES and SDF-1α. The remaining cytokines included six growth factors (G-CSF, GM-CSF, HGF, LIF, VEGF, β-NGF) and two immunoregulatory cytokines (IL-6, TNF-α) (Table S1).

### 3.4. Common Indicated Cytokines in Breast Cancer and Glioma 3D-Models

A total of twenty cytokines, exhibiting the highest log2FC, a hallmark of both BC and glioma, were subjected to comparative analysis to assess the magnitude of induction between the models (Figure 5).

Among all 3D-3 tumor models, the highest induction values were observed for LIF (log2FC = 8.69 in 3D-3HEC; 7.72 in 3D-3LEC), MCP-3 (7.16 in 3D-3LEC), and IL-8 (7.08 in 3D-3HEC) (Table S3).

The stromal component was found to induce the production of 28 cytokines in the breast cancer model and 15 cytokines in the glioma model. Cross-sectional analysis of these two subsets revealed 14 cytokines common to both tumor models: G-CSF, GM-CSF, GRO-α, HGF, IL-6, IP-10, LIF, MCP-1, MIP-1α, MIP-1β, RANTES, SDF-1α, TNF-α, and β-NGF (Figure 6).

#### 3.4.1. Functional Composition of the Cross-Tumor Cytokine Intersection

Of the fourteen cytokines that are common to both breast cancer and glioma, seven are chemokines (GRO-α, IP-10, MCP-1, MIP-1α, MIP-1β, RANTES, SDF-1α), five are growth factors (G-CSF, GM-CSF, HGF, LIF, β-NGF), and two possess immunoregulatory properties (IL-6 and TNF-α). Consequently, the chemokine component predominates in the cross-tumor response to stromal addition, suggesting the activation of chemokine signaling in the tumor microenvironment of both heterotypic models.

#### 3.4.2. Contribution of BC Cells to the Cross-Tumor Intersection

Of the 14 cytokines found in both breast cancer and glioma, only three were present in all three breast cancer cell lines, including MDA-MB-231. The remaining 11 shared cytokines were detected in the MCF7 and SK-BR-3 lines, but not in the MDA-MB-231 line. Therefore, HGF, LIF, and MCP-1 are the core cytokines characteristic of all the breast cancer subtypes examined.

#### 3.4.3. Annotation of Cytokines Shared Across Both Tumor Types

Among the cytokines common to breast cancer and glioma, nine (G-CSF, GRO-α, IL-6, IP-10, LIF, MIP-1α, RANTES, TNF-α, and β-NGF) exhibited an “emergent” secretion pattern, whereas five (GM-CSF, HGF, MCP-1, MIP-1β, and SDF-1α) were classified as “stroma-contributing”.

In the context of breast cancer, eight of the fourteen shared cytokines (G-CSF, GM-CSF, GRO-α, HGF, IL-6, LIF, MCP-1, MIP-1β) were classified as “stroma-contributing” in all comparisons, while the remaining six (IP-10, MIP-1α, RANTES, SDF-1α, TNF-α, β-NGF) showed an “emergent” secretion pattern in at least one comparison. In the glioma model, nine of the fourteen shared cytokines (64%) showed an “emergent” component, in contrast to six of the fourteen (43%) in the breast cancer model. This difference suggests that the shared secretion pattern may arise through different routes in the two models. In glioma, the “emergent” pattern was predominant, whereas in breast cancer a larger proportion of the shared cytokines was classified as “stroma-contributing”.

### 3.5. Suppression of Secretion

The suppression of cytokine secretion, as defined by the criteria log2FC ≤ −1 and upper bound of the 95% CI < 0, was observed in 11 out of 256 comparisons, amounting to 4.3% of the total. The suppression patterns are illustrated in the form of heatmaps (see Figure 2 and Figure 4), in which the suppressed cytokines are denoted by a “v” symbol.

Suppression of b-FGF was detected in six of eight comparisons, the most frequent decrease among all cytokines analyzed. In the context of breast cancer, the expression of b-FGF was found to be suppressed in the 3D-3LEN (log2FC = −2.99), 3D-3LEC (−1.19), 3D-3HEN (−2.87), 3D-3TEN (−3.82), and 3D-3TEC (−3.04) models (Table S3). However, no significant alterations were observed in the 3D-3HEC model (log2FC = −0.27).

In the context of glioma, the suppression of b-FGF was observed upon the addition of microglia to the 3D-2GA model (log2FC = −1.28), with a concomitant trend toward suppression upon the addition of astrocytes to the 3D-2GM model (log2FC = −0.89). All cases of decreased b-FGF secretion were annotated as “emergent”, meaning that the level detected in the stromal monoculture was lower than that in the corresponding 3D-2 culture. This pattern may be consistent with a decrease in b-FGF secretion upon the addition of stromal cells rather than with a dilution effect. Decreased b-FGF secretion was observed in both breast cancer and glioma models, which may point to a shared feature of FGF-related signaling in these systems.

MDA-MB-231 was the sole cell line in which GM-CSF (log2FC = −1.26 for the 3D-3TEN model; −2.07 for 3D-3TEC) and IP-10 (−2.87 for 3D-3TEN; −1.71 for 3D-3TEC) were suppressed (Table S3).

In contrast, models based on the two other breast cancer lines showed elevated secretion of these cytokines. The decreased secretion of GM-CSF and IP-10 in MDA-MB-231 models, together with the limited number of changes observed, may point to a subtype-specific reorganization of the cytokine secretome in triple-negative breast cancer. In this setting, the addition of stromal cells coincided with fewer increased cytokines and with decreased levels of specific signals.

Weak suppression of IL-1ra (log2FC = −1.02) was seen in one case, when astrocytes were added to the 3D-2GM model, and was also classified as "emergent”.

### 3.6. Functional Analysis of Induced Cytokines

Of the 28 cytokines whose secretion was stimulated by the addition of stromal cells in breast cancer, 11 were chemokines, 7 were growth factors, and 10 were immunoregulatory cytokines. Similarly, in the context of glioma, 7 of the 15 secreted cytokines were identified as chemokines, 6 were classified as growth factors, and 2 were categorised as immunoregulatory. These preliminary findings require validation in additional glioblastoma models. Of the 14 cytokines commonly detected in both tumour types, 7 were chemokines, 5 were growth factors, and 2 were immunoregulatory cytokines. Consequently, chemokines constituted the predominant category in each individual tumor model and in the shared intersection, thereby emphasizing the pivotal role of chemokine remodeling in the interaction with the stromal microenvironment. The distribution of induced cytokines by functional class and tumor type is presented in Table 2.

## 4. Discussion

The present study employs a quantitative comparative analysis to assess the induction of cytokine secretion in 3D spheroid models of two tumor types, namely breast cancer and glioma, following the addition of stromal cells. The implementation of a uniform statistical criterion (log2FC ≥ 1, lower bound of the 95% bootstrap CI > 0) across 256 pairwise comparisons facilitated the comparison of stroma-induced cytokine programs between tumors of different histogenesis and between distinct stromal components. This uniform criterion further supports that the terms “induced” and “suppressed” are used as operational statistical classifications rather than as indications of a specific causal mechanism or cellular source.

### 4.1. CAFs Were Associated with a Broader Spectrum of Secreted Cytokines Compared to Normal Fibroblasts

In this study, BrC4f cells (CAFs) were associated with a broader spectrum of induced cytokines than normal BN120f fibroblasts (NFs). At the union level across cell lines, the set of 28 cytokines associated with CAFs was found to be linked with the set of 23 cytokines associated with NFs. Five of these cytokines were observed exclusively by CAFs. At the 2/3 consensus level (induction in at least two out of three lines), ten cytokines were identified as CAF-specific in this model (Figure 2, Table S1). The augmentation of the cytokine repertoire at the 2/3 consensus level relative to the union (10 vs. 5) is consistent with the hypothesis that CAFs may be consistent with an activated phenotype with an expanded secretory repertoire that includes pro-inflammatory and immunoregulatory factors relative to normal fibroblasts [25,26]. Transcriptomic studies have revealed substantial differences between CAFs and normal fibroblasts, with up to 818 differentially expressed mRNAs. These differences manifest as an expanded spectrum of secreted cytokines at the protein level [27].

The 3/3 consensus for BrC4f, comprising seven cytokines, includes two additional cytokines (MCP-1 and MIP-1α) compared to the BN120f consensus (HGF, IL-10, LIF, MCP-3, SCGF-β). This finding suggests that CAFs not only increase the amplitude of secretion but also broaden the range of induced chemokines, which may be pertinent for the attraction of immune cells to the tumor microenvironment [28]. Furthermore, MCP-1 expression can vary across different cancer cell lines derived from the same organ. For instance, MCP-1 expression levels are elevated in invasive breast cancer cell lines (e.g., BT594, Hs578T, and MDA-MB-231) compared to non-invasive lines (e.g., MCF7 and T47D) [29]. However, MCP-1 is secreted not only by tumor cells but also by stromal cells surrounding the tumor parenchyma [30,31]. Furthermore, studies have demonstrated a correlation between TGF-β signaling and MCP-1 expression in fibroblastic cells [32].

### 4.2. The LIF/HGF/SCGF-β Cluster and Link to Immune Evasion

The 3/3 consensus across all three breast cancer cell lines revealed a cytokine cluster comprising LIF, HGF, and SCGF-β, together with IL-10, MCP-1, MCP-3, and MIP-1α. In a closely related study that employed an identical breast cancer model system, the LIF, SDF-1, HGF, and SCGF-β cluster was identified as a potential regulator of PD-L1 expression in tumor cells [13]. It is noteworthy that SDF-1α was increased in models involving MCF7 and SK-BR-3, but not MDA-MB-231. By contrast, all three breast cancer cell lines exhibited responses to LIF, HGF, and SCGF-β.

Leukemia inhibitory factor (LIF) has attracted particular attention as a CAF-derived factor that regulates stemness and plasticity in breast cancer cells [33,34]. In triple-negative breast cancer with a high metastatic risk, LIF is expressed predominantly in FAP-positive CAFs (41.1% of cases) and promotes tumor cell migration and PD-L1 expression [34]. LIF also drives metabolic reprogramming via the AKT/GLUT1 axis, thereby facilitating tumor progression [35].

In this study, LIF showed increased secretion in all breast cancer lines (3/3 consensus), with maximal induction upon the addition of BrC4f to the 3D-2HE (log2FC = 8.69) and 3D-2LE (log2FC = 7.72) models. This is consistent with CAFs being a major contributor to LIF secretion, although bulk measurements cannot definitively identify the cellular source.

### 4.3. TNBC’s Cytokine Change and Stromal Induction

The spheroid model based on MDA-MB-231 cells (TNBC) showed the lowest number of induced cytokines, both upon the addition of BN120f (*n* = 7) and BrC4f (*n* = 8), which is 2- to 3.5-fold fewer than in models with MCF7 (16/25) or SK-BR-3 (20/27). This finding may be consistent with the documented tendency of MDA-MB-231 cells to display a pronounced mesenchymal phenotype, characterized by autonomous production of pro-inflammatory cytokines. Such autonomous secretion could potentially reduce the relative contribution of stromal-derived signals in this model [36,37]. As demonstrated by Broad et al. (2021), the presence of CAFs was associated with a protective effect against the effects of chemotherapy in TNBC cells, including the MDA-MB-231 line [38]. This protective effect is attributed to the activation of interferon signaling pathways. In that study, the blockade of IFN receptors abrogated CAF-mediated chemoprotection. High expression of IFN-β1 in CAFs was associated with diminished survival outcomes following chemotherapy, particularly in claudin-low tumors, a category that encompasses MDA-MB-231 and MDA-MB-157 [38]. These findings raise the possibility that, in MDA-MB-231-based spheroid models, the presence of stromal cells may be associated with alternative cellular programs (survival, reduced chemosensitivity) rather than with a broad increase in cytokine secretion.

Of particular interest is the pronounced decrease in GM-CSF and IP-10 secretion in the 3D-3TEN (log2FC = −1.26 and −2.86, respectively) and 3D-3TEC (log2FC = −2.07 and −1.71) models. Both cytokines have been implicated in antitumor immunity: GM-CSF is involved in the recruitment and activation of dendritic cells, while IP-10 (CXCL10) contributes to the attraction of CD8^+^ T lymphocytes and NK cells [37]. The decreased secretion of these cytokines in TNBC models may be compatible with a mechanism of immune evasion, although functional validation would be required to confirm this interpretation. This finding may be consistent with data indicating that TNBC is characterized by cytokine-dependent resistance pathways involving TNF-α, IL-6, and IL-10 [39]. The results also align with observations that Notch-mediated tumor–stromal networks in TNBC selectively regulate the expression of chemokines, including CXCL8 (IL-8) [40].

### 4.4. Decreased b-FGF Secretion in the Presence of Stromal Cells

b-FGF secretion was decreased in six out of eight comparisons, and in all cases, an “emergent” pattern was observed, i.e., the level of secretion in the stromal component was lower than that in the 3D-2 culture. This observation is of interest because b-FGF is generally regarded as a pro-angiogenic factor that promotes tumor neovascularization [41,42]. However, within the context of co-culture with endothelial cells, which themselves are a source of VEGF and other angiogenic factors, the suppression of b-FGF could reflect a compensatory shift from FGF-dependent to VEGF-dependent angiogenic signaling. It has been reported that b-FGF plays a role in acquired resistance to anti-VEGF therapy [42], and in 3D glioma models, exogenous b-FGF enhances angiogenesis only under normoxia, where endogenous factor production is already sufficient [43]. The results of the present study may be compatible with an “angiogenic switch” model, in which the introduction of stromal cells shifts the balance from autocrine b-FGF signaling toward paracrine VEGF-dominated signaling. If confirmed, this would have potential implications for combined anti-angiogenic therapy [44,45].

### 4.5. Astrocytes Are More Potent Inducers than Microglia

In the glioma model based on the U87MG tumor cell line, the addition of astrocytes to the 3D-2GM culture was associated with increased secretion of 13 cytokines, of which nine were classified as “emergent”. Conversely, the addition of microglia to 3D-2GA led to an increase in the secretion of only eight cytokines, of which two were designated as “emergent”. It is imperative to note that these observations are based exclusively on the U87MG model and should be interpreted within the context of that particular model only.

The predominance of the “emergent” pattern upon astrocyte addition may indicate de novo secretion of cytokines that were absent from the corresponding stromal monoculture. This pattern could reflect paracrine signaling between cell populations, although bulk measurements cannot definitively establish the cellular source. Conversely, the majority of secreted cytokines were categorised as “stroma-contributing” following the addition of microglia, which may indicate an additional contribution from microglia to the overall secretome.

These findings may be consistent with evidence that reactive astrocytes in glioblastoma undergo transcriptional reprogramming via the JAK/STAT pathway, accompanied by the release of anti-inflammatory cytokines (TGF-β, IL-10, G-CSF) [46]. The intricate interplay between astrocytes and microglia contributes to the establishment of an immunosuppressive microenvironment [46,47]. Recent studies have demonstrated that glioma-induced astrocytes suppress tumor-specific T-cell immunity via the IL-11/STAT3/TRAIL pathway [48], and barcoded viral tracing has revealed immunosuppressive astrocyte–glioma interactions [49]. Alongside TGF-β and IL-10, G-CSF has been reported among the anti-inflammatory cytokines associated with astrocyte–microglia cooperation in the tumor microenvironment [46].

In addition to astrocytes and microglia, the glioblastoma microenvironment contains other stromal and immune populations, including macrophages and mesenchymal stromal cells. These cell types are increasingly recognized not only as modulators of tumor progression but also as potential delivery platforms for therapeutic agents [50]. Despite the absence of these components in the current model, their described secretory and immunomodulatory properties demonstrate significant overlap with the astrocyte- and microglia-associated cytokine patterns observed in this study. This overlap indicates the possibility that the identified cytokine programs may be part of a more extensive stromal communication network extending beyond the specific cell types that were examined in this study.

### 4.6. A Chemokine Scaffold Shared by Breast Cancer and Glioma Models

The intersection of cytokine secretion patterns in breast cancer (28 cytokines) and glioma (15 cytokines) models comprised 14 cytokines, with chemokines predominant among them. GRO-α, IP-10, MCP-1, MIP-1α, MIP-1β, RANTES, and SDF-1α (see Table 2). The remaining components included five growth factors (G-CSF, GM-CSF, HGF, LIF, and β-NGF) and two immunoregulatory cytokines (IL-6 and TNF-α). This predominance of chemokines suggests that immune-cell recruitment via chemokine gradients may represent a shared feature of stromal–tumor communication in the models tested here.

However, because the glioma model is based on a single cell line (U87MG), this finding should be regarded as a candidate shared feature rather than evidence of a general mechanism. This observation is in line with existing evidence on the role of chemokines in regulating the tumor microenvironment in solid tumors and gliomas [47,48,49].

The concurrent increase in IL-6 and TNF-α in both tumor types may point to shared pro-inflammatory signaling components. IL-6/STAT3 signaling has been implicated as a stromal-activated pathway in tumors of different origin [51]. TNF-α has been implicated in Notch-mediated tumor–stromal–inflammatory networks that have been described for TNBC [40].

The functional architecture of the identified cytokine core is consistent with a dual role in immune-cell trafficking and immunosuppression. MCP-1 (CCL2), MIP-1α (CCL3), and MIP-1β (CCL4) are well-characterized monocyte and macrophage chemoattractants [52], whereas IP-10 (CXCL10) and RANTES (CCL5) guide the recruitment of effector T cells and NK cells through CXCR3- and CCR5-dependent signaling [53]. It is important to acknowledge the established correlation between these chemokines and pro-inflammatory effector-cell infiltration, as opposed to the more general immunosuppression [54]. However, the concurrent secretion of these chemokines alongside IL-6, LIF and IL-10 indicates that the recruited immune cells encounter a microenvironment that favours their polarisation towards a pro-tumourigenic or exhausted phenotype, rather than productive anti-tumour effector function. IL-6 and IL-10 have been shown to promote immunosuppressive, M2-like macrophage polarization and T-cell exhaustion [55], while LIF, acting through the gp130/JAK–STAT3 axis, has been demonstrated to drive macrophage-mediated immunosuppression and attenuate T-cell activity [56]. This interpretation aligns with the emerging view that chemokine gradients in solid tumors frequently contribute to immune-cell retention in the stromal compartment, the immune-excluded phenotype, rather than to effective tumor infiltration [57], a concept that is broadly consistent with current models of chemokine-driven organization of the tumor microenvironment [58].

### 4.7. Operational Classification of Induced Cytokines 

The classification of induced cytokines into “emergent” and “stroma-contributing” categories should be interpreted as an operational distinction rather than direct evidence of cellular origin. Bulk measurement of these factors precludes the ability to attribute the production of cytokines to a particular cell population. The phenomenon of “emergent” induction may be indicative of paracrine signaling. However, it is plausible that this phenomenon may also result from alterations in proliferation, survival, or nonlinear additive effects within the composite culture.

Moreover, the transition from two-component to three-component cultures has been shown to result in alterations in both the total cell number and the relative proportions of cell populations. Therefore, elevated cytokine levels in 3D-3 models should not be taken as definitive proof of paracrine regulation. These factors may also be indicative of additive secretory contributions, altered population ratios, or indirect effects on proliferation and survival.

In the glioma model, the addition of astrocytes was predominantly associated with “emergent” cytokines (9/13), while the addition of microglia was primarily associated with “stroma-contributing” ones (6/8). Among the fourteen cytokines shared by both models, nine exhibited an “emergent” secretion profile in glioma compared with six in breast cancer. This difference may indicate more pronounced paracrine signaling in the glioma model, although it cannot be confirmed by bulk measurements alone. Should subsequent research substantiate the notion that “emergent” induction is indicative of paracrine signaling, this distinction may hold therapeutic significance. The blockade of a signaling pathway such as JAK/STAT could potentially suppress induction, while a stroma-contributing pattern would likely necessitate direct targeting of stromal secretory function. However, it should be noted that this interpretation remains provisional, as the current measurements are bulk-level [46].

### 4.8. Cytokine Secretion Patterns in Other 3D Tumor Models

The present model differs from several previously described 3D tumor spheroid systems in both scope and analytical strategy. Berens et al. (2015) established a hanging-drop spheroid assay to quantify cancer cell invasion into a 3D matrix, while Wagner and Koyasu (2020) developed a melanoma spheroid model co-cultured with immune cells to study tumor–immune interactions [59,60]. Sant and Johnston (2017) reviewed methods for generating uniform, HTS-compatible spheroids for cancer drug discovery [61]. These studies primarily address methodological standardization, invasion assessment, or pharmacological screening. In contrast, the present study uses the 3D spheroid platform to quantify changes in cytokine secretion associated with the addition of stromal cells and to compare these changes between two tumor models within a single statistical framework. The classification of induced cytokines as “emergent” or “stroma-contributing” provides an additional layer of information about the secretion pattern that is not captured by conventional fold-change analyses or by the invasion- and drug-screening-oriented readouts of the aforementioned models.

The patterns of increased cytokine secretion observed upon addition of a stromal component, and the presence of a shared chemokine component, are in line with a growing body of data from 3D models of other tumor types.

A comparative analysis of the obtained results with these models allows for an evaluation of the universality and specificity of the identified programs.

In the study with the most similar design, Bersano et al. (2023) analyzed 45 cytokines in 20 colorectal cancer (CRC) lines in 2D, 3D spheroid, and co-culture formats with NFs and CAFs [62]. Consistent with the findings of our study, the addition of fibroblasts was associated with an increase in total cytokine secretion in the majority of lines. Furthermore, NFs and CAFs exhibited similar patterns of response, with approximately 70% of cytokines demonstrating altered expression, independent of fibroblast type. A set of pro-inflammatory, pro-angiogenic, and EMT-associated factors, including EGF, HGF, PIGF-1, GM-CSF, IL-5, IL-9, and IL-6, was secreted exclusively in 3D and co-culture. LIF and HGF were identified as the primary discriminators of “high-secreting” and “low-secreting” lines [62]. These factors overlap with the LIF/HGF/SCGF-β cluster identified in the breast cancer and glioma models in our study. The incorporation of macrophages in triple culture was associated with increased levels of MCP-1 (CCL2), MIP-1α (CCL3), and IL-1Rα, together with decreased levels of VEGF-A and SDF-1α. Furthermore, a shift in polarization toward M1 was observed, as indicated by an increase in CCL3, while NFs promoted M2, as indicated by an increase in CCL2 [62]. The observed overlap in MCP-1, MIP-1α, SDF-1α, HGF, GM-CSF, IL-6, and LIF with the cross-tumor core identified in our study may point to common elements of the stromal cytokine response across CRC, BC, and glioma.

As demonstrated by Koppensteiner et al. (2025), when exposed to T-cell effector cytokines IFN-γ and TNF-α, CAFs from non-small cell lung cancer (NSCLC) transition to an iCAF-like inflammatory secretory profile [63]. This switch is accompanied by increased production of the following: IL-6, LIF, IL-33, GM-CSF, IL-1ra, chemokines (CCL2, CCL3, CCL4, CCL20, and CXCL8), and Th1-characteristic chemokines (CCL9/CCL10/CCL11). The latter are ligands for CXCR3 and play a critical role in the recruitment of effector CD8^+^ T cells. In contrast, the secretion of CXCR12 (SDF-1α) was suppressed by IFN-γ [63]. This study complements our observations at the functional level. The factors IP-10 (CXCL10), MCP-1 (CCL2), MIP-1α/β (CCL3/CCL4), and SDF-1α are part of the shared core identified in our study, and the divergent regulation of CXCL9–11 (up) and CXCL12 (down) reported by Koppensteiner et al. indicates that this chemokine component is not monolithic but may represent a balance of pro- and anti-recruitment signals depending on the stromal stimulus.

In a co-culture model of patient-derived organoids of pancreatic ductal adenocarcinoma (PDAC) with macrophages, a Macrophage/CCL5/Sp1/AREG feedback loop was identified. Macrophage-derived CCL5 (RANTES) was found to activate the CCR5/AKT/Sp1/CD44 axis, thereby conferring stemness and gemcitabine resistance to tumor cells. In addition, tumor-derived AREG was found to stimulate CCL5 secretion by macrophages via the Hippo-YAP pathway [64]. CCL5 was also identified in the shared core of the breast cancer and glioma models, which may point to its role as a mediator of stromal–tumor communication. The PDAC data link this chemokine to clinically relevant drug resistance, which may support the translational relevance of our observation.

In a study of squamous cell carcinoma of the head and neck, Francois et al. (2026) used a triple culture (FaDu cells + fibroblasts + U937 macrophages), in which the immunosuppressive M2 phenotype of macrophages with overexpression of CD163, PD-L1, IL-10 and CCL2 predominated [65]. Subsequent photodynamic therapy reprogrammed macrophages toward an antitumor M1 phenotype, as detected by increased CD80 and decreased CD206. This reprogramming occurred specifically in the presence of fibroblasts. The role of CCL2 (MCP-1) in monocyte recruitment and M2 polarization is consistent with the presence of MCP-1 in the shared core of the breast cancer and glioma models. This may support the view that stroma-mediated M2 polarization is a recurrent feature of 3D tumor models.

Zymantaite et al. (2026) reported that cultivation of ovarian cancer cell lines together with CAFs or WS1 fibroblasts led to an increase in spheroid formation in cell lines that did not form spheroids in monoculture, as well as an increase in the compactness of these structures [66]. These outcomes are consistent with those reported in our previous studies [12,13]. The 3D spheroids in that study exhibited elevated expression of stemness/EMT and angiogenesis genes compared with 2D cultures. While Zymantaite et al. focused on morphology and gene expression, their findings are in line with our observation that the stromal component is associated with a qualitative change in the tumor model rather than only with an additive contribution to the existing secretome.

It is also noteworthy to mention the work of Babazadeh et al. (2021), which demonstrated that mesenchymal stem cells directly induce M2 macrophage polarization and stimulate tumor growth through the secretion of CXCL12 (SDF-1α) [67]. This finding may provide a context for the presence of SDF-1α in the shared core identified in our study. SDF-1α has been reported to function not only as a chemoattractant but also as a polarizing signal, which may link the stromal compartment to immunosuppressive reprogramming of myeloid cells.

Across the models reviewed here, the addition of a stromal component, including fibroblasts and/or macrophages, was consistently associated with increased secretion of a set of cytokines that overlaps with the core identified in our study: MCP-1 (CCL2), RANTES (CCL5), SDF-1α (CXCL12), IP-10 (CXCL10), MIP-1α/b, IL-6, HGF, LIF, and GM-CSF. These observations support the hypothesis that the chemokine-dominated core identified here may represent a shared component of stromal–tumor communication. However, additional glioma models and independent breast cancer cohorts are required before this can be considered a broadly conserved, histogenesis-independent mechanism. At the same time, differences between models, for example, the divergent regulation of CXCL9–11 versus CXCL12 in NSCLC, or the association between CCL5 and chemoresistance in PDAC, may reflect context-specific branches of this shared component.

## 5. Study Limitations

The present study has several limitations. Firstly, the experimental design was configured with three biological replicates per group, a configuration that offers minimal statistical power. Bootstrap estimation of confidence intervals was utilized as a descriptive tool to assess variability within the available data. This approach does not enhance statistical power and is unable to compensate for the limited number of biological replicates. Consequently, the interpretation of induction calls should be approached with a degree of caution. It should be noted that no correction for multiple comparisons (256 tests) was applied. Conversely, a consensus approach (induction in ≥2 of 3 cell lines) was employed as a biological filter for false-positive results. The validation of the key findings (3/3 consensus, cross-tumor intersection) is planned to be performed in an independent cohort or with an increased number of biological replicates.

Secondly, the model does not incorporate immune cells (T lymphocytes, macrophages), which limits the interpretation of the immunoregulatory function of the secreted cytokines. The incorporation of an immune component into 3D-4 cultures will enable the validation of the functional consequences of the identified cytokine programs [68].

Thirdly, the use of immortalized cell lines and simplified 3D spheroids may not fully recapitulate the complexity of primary tumors. It is important to note that cell lines can undergo genetic and phenotypic drift. In contrast, spheroids lack the native extracellular matrix, vascular networks, and mechanical cues characteristic of tumor tissue in vivo. Consequently, the absolute cytokine levels and response amplitudes observed in this study should be interpreted in the context of the specific model employed. However, identical experimental conditions improve comparability between the two models but do not exclude the possibility of shared or model-dependent in vitro artifacts. Therefore, the observed comparative patterns and the identified shared cytokine set should be interpreted with this caveat in mind. The utilization of patient-derived organoids or tissue explants would constitute a significant subsequent step in validating these findings within more clinically pertinent contexts [69].

Fourthly, the analysis was constrained to the 32 cytokines of the xMAP/Bio-Plex panel, thus failing to encompass the complete secretome. To obtain a more comprehensive overview, it is necessary to expand the panel and move toward proteomic secretome analysis.

Finally, the glioma model of this study is based on a single cell line (U87MG). In light of the well-documented molecular and phenotypic heterogeneity of glioblastoma, it is imperative to exercise caution when generalizing the identified cytokine patterns to all gliomas. In order to assess the robustness of the findings, it is necessary to validate the results in at least one additional glioblastoma cell line, preferably with a distinct molecular background.

## 6. Perspectives

The shared set of 14 cytokines identified in this study may represent a candidate target for further investigation. The chemokines MCP-1, SDF-1α, and RANTES, which are part of this shared set, have been implicated in immune-cell recruitment and angiogenesis [47,70]. Blocking these chemokines or their receptors may be a rational direction for future studies, although this remains to be tested.

Of particular interest is the fact that LIF was detected in all three breast cancer cell lines and in the glioma model. LIF inhibitors such as EC359 have been reported to reduce migration and PD-L1 expression in TNBC, which makes LIF a candidate for further investigation in combination immunotherapy [34].

The decreased secretion of GM-CSF and IP-10 in MDA-MB-231-based models may suggest a potential approach for modulating antitumor immunity in TNBC, for example, through exogenous delivery of these cytokines or by interfering with the mechanisms responsible for their reduction, although this remains hypothetical. The observed shift from b-FGF to VEGF upon stromal addition could provide a rationale for combined anti-angiogenic strategies targeting both pathways, should this pattern be confirmed in further models [42,45].

The HGF/c-MET axis has been implicated in resistance to tyrosine kinase inhibitors, particularly lapatinib and neratinib. Comparison with the data of Bersano et al. (2023) raises the possibility that HGF secretion in the stromal niche could serve as a biomarker of primary insensitivity to anti-EGFR therapy, although this hypothesis requires dedicated validation [62,71]. In the aforementioned study, HGF and LIF served as discriminators of “high-secreting” colorectal cancer lines.

The shared cytokine set may be relevant for understanding how an immunosuppressive microenvironment is established as a barrier to immunotherapy. One constituent of the core, CXCL10 (IP-10), functions as a ligand for CXCR3, thereby attracting effector T cells [64]. Another component, SDF-1α (CXCL12), was reported by Koppensteiner et al. (2025) to be decreased by IFN-γ while being maintained under basal conditions [63]. Collectively, these observations raise the possibility of a dual functional circuit. In the absence of therapeutic intervention, CXCL12 may predominate, potentially promoting M2 macrophage polarization and angiogenesis. Upon immune activation, CXCL9/10/11 expression may increase, which in principle could enhance CD8+ T-cell infiltration. However, the Koppensteiner model indicates that this switch is accompanied by the production of IL-6, LIF, and GM-CSF [63]. Given the reported role of IL-6 and LIF in inducing PD-L1 expression via the STAT3 pathway, it is conceivable that therapy-induced inflammatory remodeling of the stroma could create a “trap” situation. In this scenario, the recruitment of T cells and the production of immunosuppressive molecules would occur simultaneously, which could potentially lead to the suppression of T-cell function. If confirmed, this would argue for combining inhibitors of chemokine receptors (e.g., CXCR4) with blockers of the IL-6/LIF axis in future studies.

The most immediate functional follow-up would involve selective blockade of one or two components of the shared core in 3D-3 models. Based on the consistency and amplitude of the secretion pattern, MCP-1, SDF-1α, and LIF represent rational first candidates. Neutralizing antibodies or small-molecule inhibitors targeting these factors would allow assessment of their contribution to tumor cell behavior and stromal communication without altering the overall architecture of the model.

## 7. Conclusions

The present study shows that the addition of stromal cells to 3D tumor models predominantly enhances cytokine secretion, and that the observed changes depended on both tumor type and stromal cell identity. In the breast cancer model, cancer-associated fibroblasts coincided with a broader array of cytokines than normal fibroblasts. Triple-negative breast cancer cells showed limited changes, with lower secretion of GM-CSF and IP-10. In the glioma model, astrocytes and microglia displayed distinct patterns, with astrocytes predominantly linked to “emergent” cytokines, defined operationally as cytokines absent from the corresponding stromal monoculture.

Despite these differences, a shared set of 14 cytokines, predominantly comprising chemokines, was identified across the breast cancer and glioma models. This set should be regarded as a candidate shared signature of stromal–tumor communication rather than definitive evidence of a universal mechanism, given the limited number of cell lines examined.

In summary, the study provides a quantitative framework for comparing changes in the secretome associated with the addition of stromal cells and identifies specific cytokine candidates for future functional validation in more complex models.

## Figures and Tables

**Figure 1:**
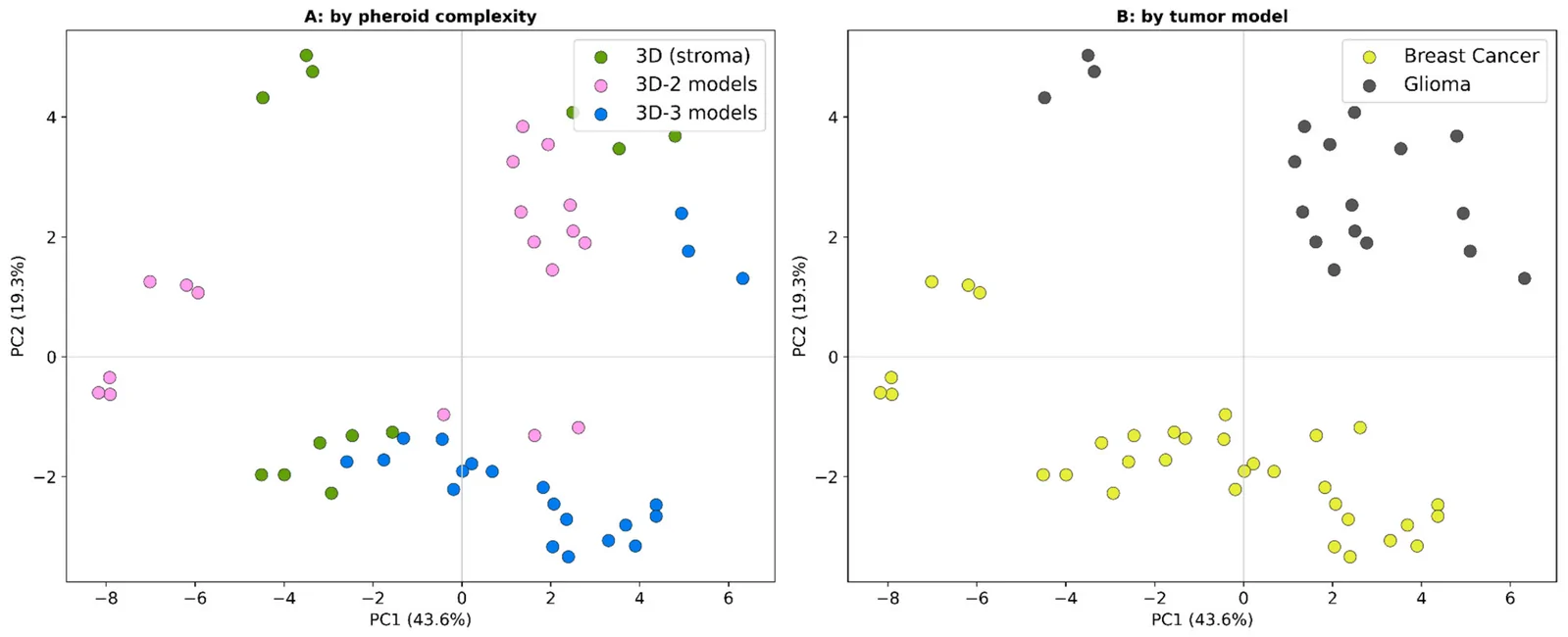
PCA of cytokine secretion profiles. Principal component analysis (PCA) applied to log-transformed (log2(value + 1)) and standardized (z-score) concentrations of 32 cytokines. (**A**) Samples are color-coded by culture complexity: stromal component (green), 3D-2 cultures (pink), 3D-3 cultures (blue). (**B**) Samples are color-coded by tumor model: breast cancer (yellow), glioma (gray). Axes represent the first two principal components, with the proportion of explained variance indicated. Experiments employed three biological replicates per group (*n* = 3).

**Figure 2:**
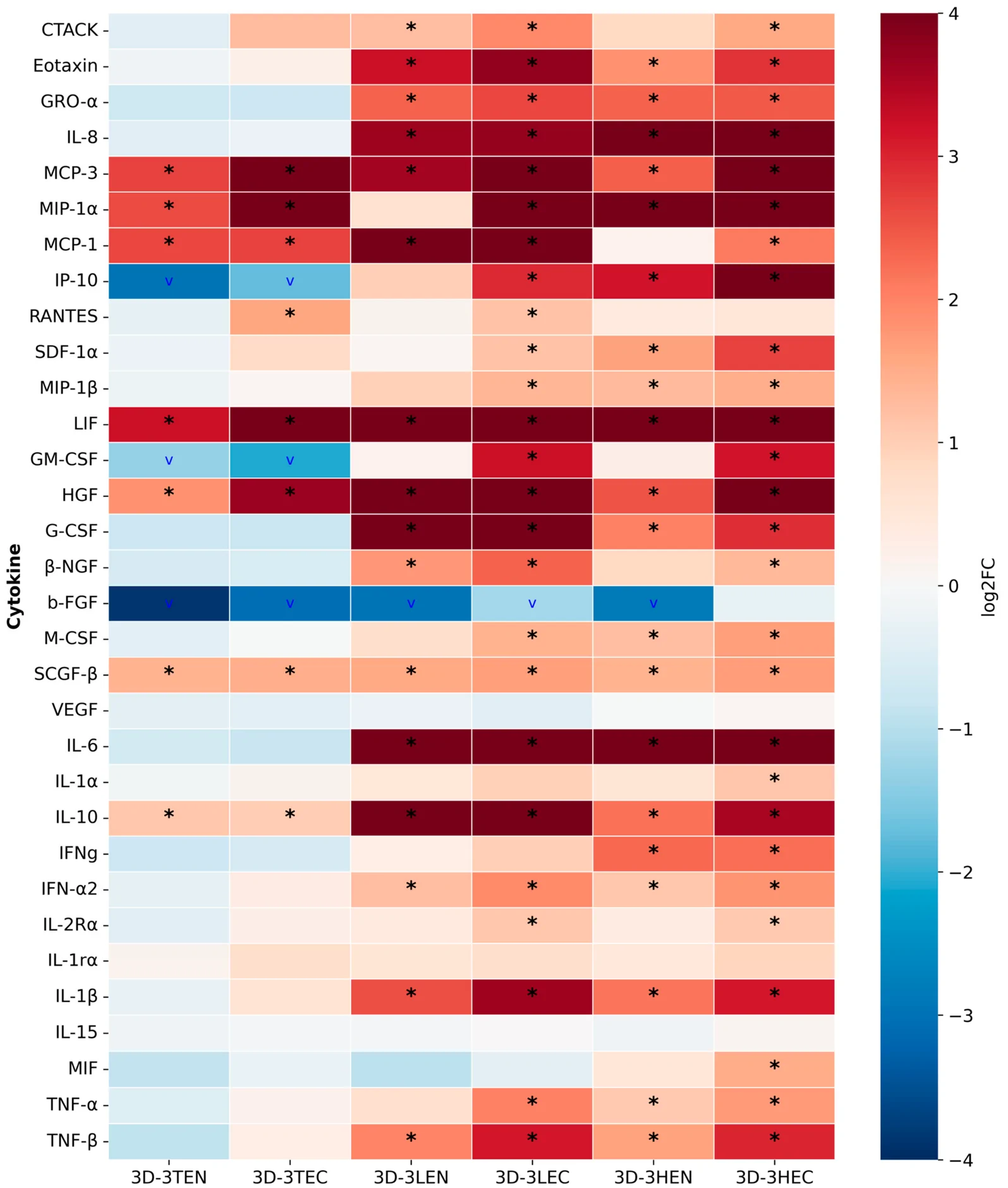
Heatmap of log2FC for breast cancer. Changes in cytokine secretion (log2FC) upon transition from 3D-2 to 3D-3 culture in the breast cancer model. The 32 cytokines are grouped by selected functional classes: chemokines, growth factors, immunoregulatory cytokines. Color scale: red—induction; blue—suppression (range from −4 to +4 log2FC). (*)—induced cytokines (log2FC ≥ 1, lower bound of 95% bootstrap CI > 0); (v)—suppressed cytokines (log2FC ≤ −1, upper bound of 95% CI < 0). *n* = 3 per group.

**Figure 3:**
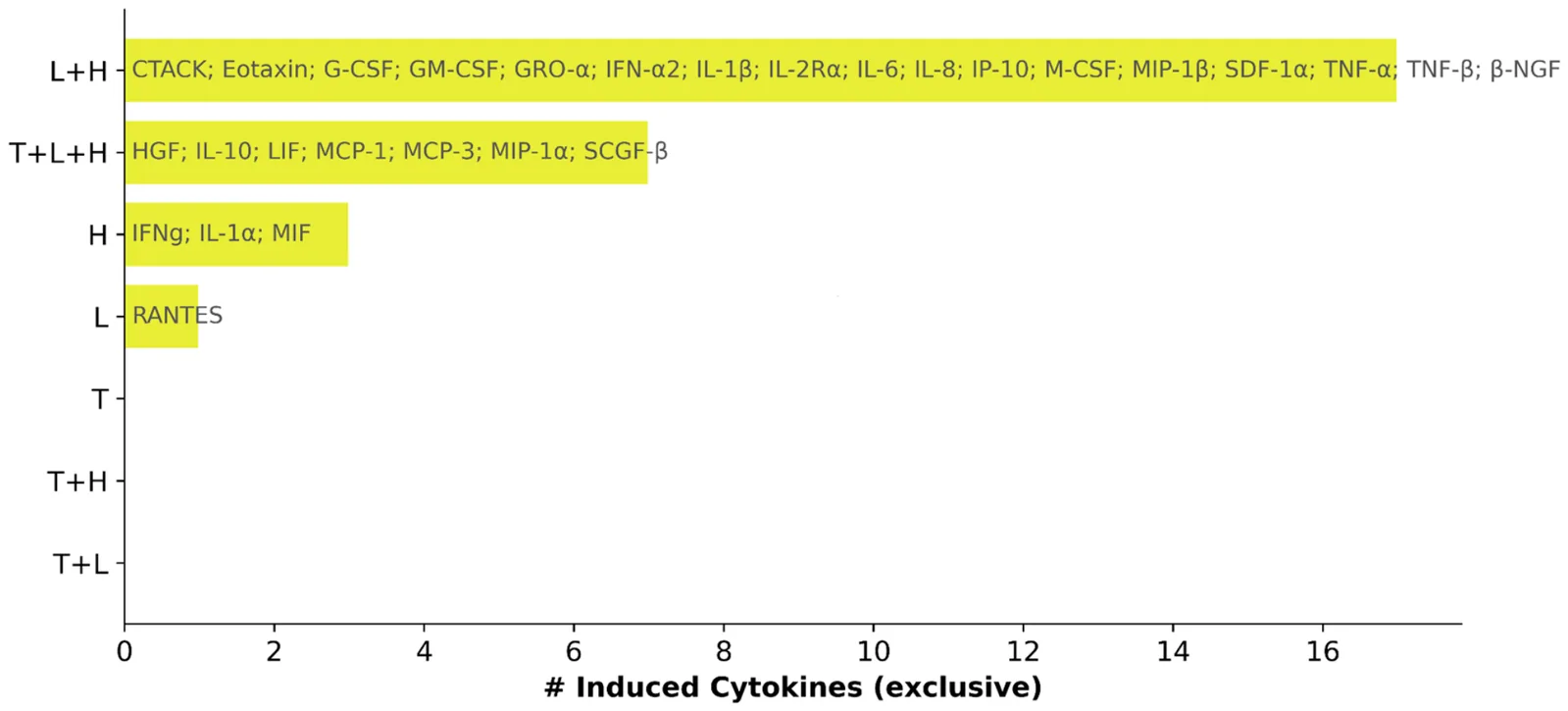
Intersections of induced cytokines across breast cancer cell lines. Intersection of the sets of induced cytokines among the three breast cancer cell lines (H = SK-BR-3; L = MCF7; T = MDA-MB-231), pooled across both fibroblast types BN120f and BrC4f. Each bar represents a combination of cell lines; bar height indicates the number of cytokines induced exclusively in that combination (excluding cytokines induced in other lines). Cytokine names are shown on the bars. *n* = 3 per group.

**Figure 4:**
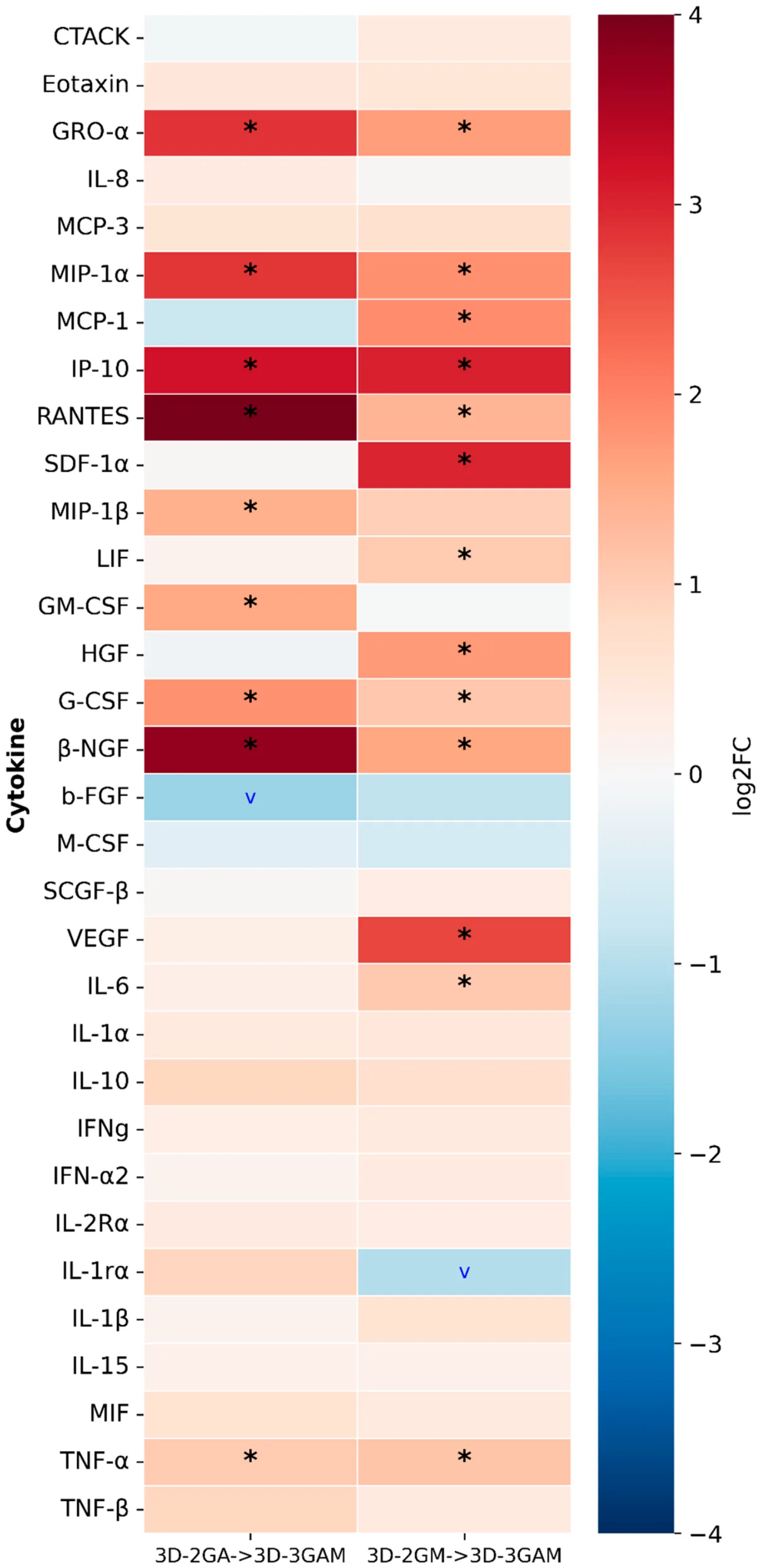
Heatmap of log2FC for glioma. Changes in cytokine secretion (log2FC) upon transition from 3D-2 to 3D-3 culture in the glioma model. Rows—32 cytokines (grouped by functional classes). Columns—two comparisons: 3D-2GA → 3D-3GAM (addition of HMC3 microglia); 3D-2GM → 3D-3GAM (addition of CCF-STTG1 astrocytes). Color scale: red—induction; blue—suppression (range from −4 to +4 log2FC). (*)—induced cytokines (log2FC ≥ 1, lower bound of 95% bootstrap CI > 0); (v)—suppressed cytokines (log2FC ≤ −1, upper bound of 95% CI < 0). *n* = 3 per group.

**Figure 5:**
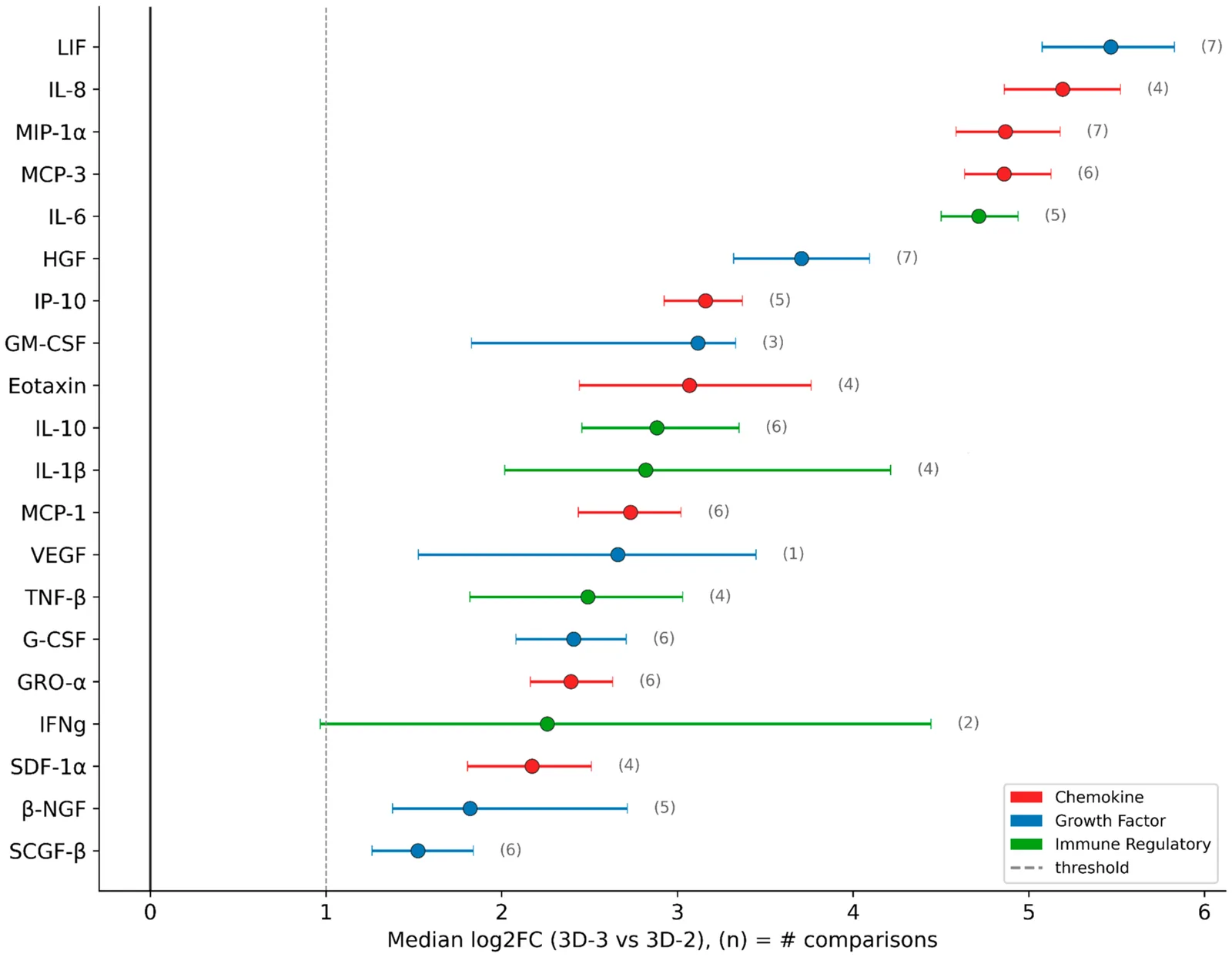
Forest plot of the induced cytokines. Twenty cytokines with the highest median log2FC across all induced comparisons (breast cancer and glioma combined). Dots—median of log2FC bootstrap distribution. Horizontal bars—95% confidence interval (*n* = 1000 iterations). Color indicates functional class: red—chemokines; blue—growth factors; green—immunoregulatory cytokines. The number in parentheses to the right of each bar indicates the number of comparisons in which the cytokine was induced. The dashed line marks the log2FC = 1 threshold. *n* = 3 per group.

**Figure 6:**
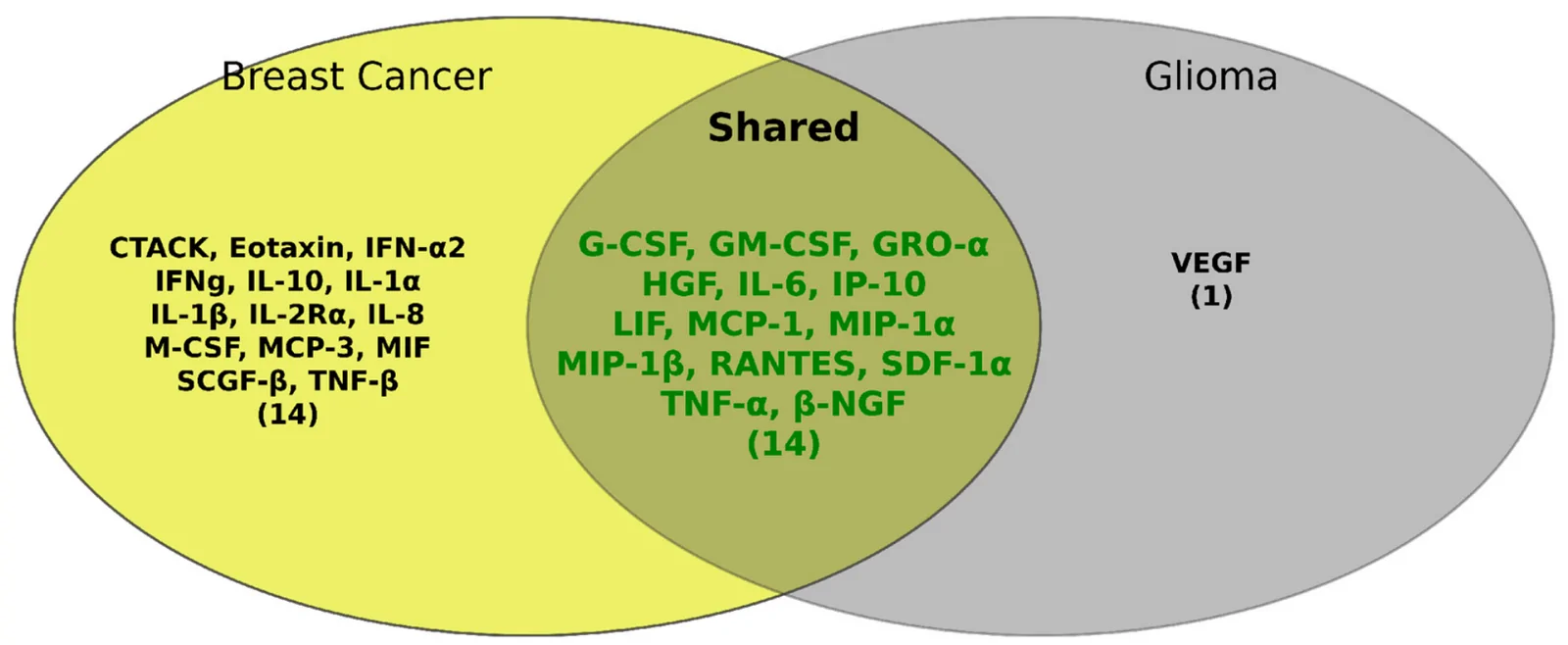
Cross-tumor intersection of induced cytokines. The Venn diagram illustrates the intersection of the sets of induced cytokines between breast cancer (union across three cell lines and two stromal types; 28 cytokines) and glioma (union across stromal additions, HMC3 and CCF-STTG1; 15 cytokines). The yellow circle indicates breast cancer, the gray circle indicates glioma, and the intersection area indicates 14 cytokines shared by both tumor models. The list of shared cytokines is located in the center. *n* = 3 per group.

**Table 1:** Description of cell cultures and 3D models.

Culture Type	Cell Line	Code	Molecular/Functional Status	Model ID
*Breast cancer*				
Cancer	SK-BR-3	H	HER2-positive	–
Cancer	MCF7	L	Luminal A	–
Cancer	MDA-MB-231	T	Triple negative	–
Endothelial	TIME	E	Microvascular endothelium	–
Stromal (normal)	BN120f	N	Normal breast fibroblasts	3D-1N
Stromal (cancer)	BrC4f	C	Breast cancer-associated fibroblasts	3D-1C
Bi-culture	SK-BR-3 + TIME	–	–	3D-2HE
Bi-culture	MCF7 + TIME	–	–	3D-2LE
Bi-culture	MDA-MB-231 + TIME	–	–	3D-2TE
Tri-culture	SK-BR-3 + TIME + BN120f	–	–	3D-3HEN
Tri-culture	MCF7 + TIME + BN120f	–	–	3D-3LEN
Tri-culture	MDA-MB-231 + TIME + BN120f	–	–	3D-3TEN
Tri-culture	SK-BR-3 + TIME + BrC4f	–	–	3D-3HEC
Tri-culture	MCF7 + TIME + BrC4f	–	–	3D-3LEC
Tri-culture	MDA-MB-231 + TIME + BrC4f	–	–	3D-3TEC
*Glioma*				
Cancer	U87MG	G	Glioblastoma	3D-1G
Stromal	CCF-STTG1	A	Astrocytes	3D-1A
Stromal	HMC3	M	Microglia	3D-1M
Bi-culture	U87MG + CCF-STTG1	–	–	3D-2GA
Bi-culture	U87MG + HMC3	–	–	3D-2GM
Tri-culture	U87MG + CCF-STTG1 + HMC3	–	–	3D-3GAM

**Table 2:** Functional summary of induced cytokines.

Function	Total in Panel (*n*)	Induced in Breast Cancer	Induced in Glioma	Cross-Tumor
Chemokine	11	*n* = 11 CTACK; Eotaxin; GRO-a; IL-8; IP-10; MCP-1; MCP-3; MIP-1α; MIP-1β; RANTES; SDF-1α	*n* = 7 GRO-a; IP-10; MCP-1; MIP-1α; MIP-1β; RANTES; SDF-1α	*n* = 7 GRO-a; IP-10; MCP-1; MIP-1α; MIP-1β; RANTES; SDF-1α
Growth factor	9	*n* = 7 G-CSF; GM-CSF; HGF; LIF; M-CSF; SCGF-β; β-NGF	*n* = 6 G-CSF; GM-CSF; HGF; LIF; VEGF; β-NGF	*n* = 5 G-CSF; GM-CSF; HGF; LIF; β-NGF
Immune regulatory	12	*n* = 10 IFN-α2; IFN-γ; IL-10; IL-1α; IL-1β; IL-2Rα; IL-6; MIF; TNF-α; TNF-β	*n* = 2 IL-6; TNF-α	*n* = 2 IL-6; TNF-α

## Data Availability

The original contributions presented in this study are included in the article and the Supplementary Materials. Further inquiries can be directed to the corresponding author. All Figures and Tables were originally created by the authors specifically for this work.

## Supplementary Materials

The following supporting information can be downloaded at https://www.mdpi.com/article/10.3390/cells15191728/s1, Table S1. Induction of cytokine secretion by adding stromal cells to 3D-2 cultures in breast cancer and glioma models. Table S2. Total protein and normalization factor data in Breast Cancer and Glioma models. Table S3. Log2FC and Confidential Interval values for all cytokine comparisons in Breast Cancer and Glioma models.
