# Supplementary material for: Stromal Remodeling of the Cytokine Secretome Reveals a Chemokine Core Across Breast Cancer and Glioma 3D Cell Culture Models
Source: Cells. 2026 Sep 22;15(19):1728. doi: 10.3390/cells15191728 (PMC13664431; doi:10.3390/cells15191728)
Supplement: Supplementary file 1 [file cells-15-01728-s001.zip › cells-4511923-supplementary.pdf]

Supplementary Materials

Table S1. Induction of cytokine secretion by adding stromal cells to 3D-2 cultures in breast cancer and glioma models

Cancer model	Cell line	Added stroma	N induced	Consensus 3/3	Consensus 2/3	Union
Breast cancer	MCF7	N	16	-	-	-
	MCF7	C	25	-	-	-
	MCF7	N or C	25	-	-	-
	MDA-MB-231	N	7	-	-	-
	MDA-MB-231	C	8	-	-	-
	MDA-MB-231	N or C	8	-	-	-
	SK-BR-3	N	20	-	-	-
	SK-BR-3	C	27	-	-	-
	SK-BR-3	N or C	27	-	-	-
	**Consensus (by BN120f)**	-	N	-	5	15
	**Consensus (by BrCBrC4f)**	-	C	-	7	25
	**Consensus (by any stroma)**	-	N or C	-	7	25
Glioma	U87MGMG	M	8	-	-	-
	U87MGMG	A	13	-	-	-
	**Union (by stroma)**	-	M or A	-	-	-
Cross-tumor intersection	-	-	-	-	-	14

Notes:

Consensus 3/3 - cytokines induced in all three breast cancer cell lines; *consensus 2/3* - induced in at least two lines; *union* - induced in at least one line. Induction criterion: log2FC ≥ 1 (twofold increase upon transition from 3D-2 to 3D-3 culture) and the lower bound of the 95% bootstrap confidence interval (n = 1000 iterations) exceeding 0. Biological replicates per group, n = 3. (-) value not applicable for the given row.

Breast cancer consensus 3/3 (any stroma), 7 cytokines: HGF, IL-10, LIF, MCP-1, MCP-3, MIP-1α, SCGF-β.

Breast cancer consensus 3/3 for BN120f, 5 cytokines: HGF, IL-10, LIF, MCP-3, SCGF-β.

Breast cancer consensus 3/3 for BrCBrC4f, 7 cytokines: HGF, IL-10, LIF, MCP-1, MCP-3, MIP-1α, SCGF-β.

Breast cancer union (any line, any stroma), 28 cytokines: CTACK, Eotaxin, G-CSF, GM-CSF, GRO-α, HGF, IFN-α2, IFN-γ, IL-10, IL-1α, IL-1β, IL-2Rα, IL-6, IL-8, IP-10, LIF, M-CSF, MCP-1, MCP-3, MIF, MIP-1α, MIP-1β, RANTES, SCGF-β, SDF-1α, TNF-α, TNF-β, β-NGF.

Glioma union (across stromal additions), 15 cytokines: G-CSF, GM-CSF, GRO-α, HGF, IL-6, IP-10, LIF, MCP-1, MIP-1α, MIP-1β, RANTES, SDF-1α, TNF-α, VEGF, β-NGF.

Cross-tumor intersection, 14 cytokines: G-CSF, GM-CSF, GRO-α, HGF, IL-6, IP-10, LIF, MCP-1, MIP-1α, MIP-1β, RANTES, SDF-1α, TNF-α, β-NGF.

Table S2. Total protein and normalization factor data in Breast Cancer and Glioma models

Group	Total protein (mg/L)			Normalization factor (k_i)		
	#1	#2	#3	#1	#2	#3
3D-1N	101,12	114,91	128,7	1,005668821	1,14281452	1,279960219
3D-1C	100,87	114,62	128,37	1,003182496	1,139930383	1,27667827
3D-2LE	106,96	121,55	136,14	1,063749378	1,208851318	1,353953257
3D-3LEN	109,16	124,04	138,92	1,08562904	1,233615117	1,381601193
3D-3LEC	109,57	124,51	139,45	1,089706614	1,238289408	1,386872203
3D-2TE	107,17	121,78	136,39	1,065837892	1,211138737	1,356439582
3D-3TEN	100,55	114,26	127,97	1	1,136350075	1,272700149
3D-3TEC	104,41	118,65	132,89	1,038388861	1,180009945	1,321631029
3D-2HE	106,07	120,53	134,99	1,054898061	1,198707111	1,342516161
3D-3HEN	108,63	123,44	138,25	1,080358031	1,227647936	1,374937842
3D-3HEC	107,04	121,63	136,23	1,064545002	1,209646942	1,354848334
3D-1A	113,17	128,6	144,03	1,125509697	1,278965689	1,432421681
3D-1M	113,35	128,8	144,25	1,127299851	1,280954749	1,434609647
3D-2GA	113,5	128,97	144,44	1,128791646	1,28264545	1,436499254
3D-2GM	113,61	129,1	144,59	1,129885629	1,283938339	1,437991049
3D-3GAM	112,04	127,32	142,6	1,114271507	1,266235704	1,418199901

Notes:

Reference group for coefficients: 3D-3TEN (coefficient = 1.0).

Total protein was measured in spheroid lysates from parallel cultures under identical conditions (one measurement per group).

Cytokine concentrations (pg/mL) were normalized to total protein by dividing by the group-specific normalization factor.

Table S3. Log2FC and Confidential Interval values for all cytokine comparisons in Breast Cancer and Glioma models

Model	Cell line	Stroma type	Comparison	Cytokine	Function	log2FC	CIlow	CI high	Effect	Annotation
BC	MDA-231	BN120f	3D-2TE -> 3D-3TEN	CTACK	Chemokine	-0.35	-0.84	0.266	unchanged	emergent
BC	MDA-231	BN120f	3D-2TE -> 3D-3TEN	Eotaxin	Chemokine	-0.074	-0.344	0.206	unchanged	emergent
BC	MDA-231	BN120f	3D-2TE -> 3D-3TEN	b-FGF	Growth factor	-3.82	-4.509	-2.87	suppressed	emergent
BC	MDA-231	BN120f	3D-2TE -> 3D-3TEN	G-CSF	Growth factor	-0.669	-1.389	0.412	unchanged	emergent
BC	MDA-231	BN120f	3D-2TE -> 3D-3TEN	GM-CSF	Growth factor	-1.26	-1.753	-0.629	suppressed	emergent
BC	MDA-231	BN120f	3D-2TE -> 3D-3TEN	GRO-a	Chemokine	-0.635	-0.942	-0.147	unchanged	emergent
BC	MDA-231	BN120f	3D-2TE -> 3D-3TEN	HGF	Growth factor	1.927	1.603	2.285	induced	stroma-contributing
BC	MDA-231	BN120f	3D-2TE -> 3D-3TEN	IFN-alpha2	Immune regulatory	-0.191	-0.473	0.097	unchanged	emergent
BC	MDA-231	BN120f	3D-2TE -> 3D-3TEN	IFNg	Immune regulatory	-0.626	-0.985	-0.269	unchanged	emergent
BC	MDA-231	BN120f	3D-2TE -> 3D-3TEN	IL-1a	Immune regulatory	-0.026	-0.325	0.245	unchanged	emergent
BC	MDA-231	BN120f	3D-2TE -> 3D-3TEN	IL-1b	Immune regulatory	-0.201	-0.612	0.271	unchanged	emergent
BC	MDA-231	BN120f	3D-2TE -> 3D-3TEN	IL-1ra	Immune regulatory	0.216	-0.071	0.555	unchanged	stroma-contributing
BC	MDA-231	BN120f	3D-2TE -> 3D-3TEN	IL-2Ralpha	Immune regulatory	-0.335	-0.595	-0.031	unchanged	emergent
BC	MDA-231	BN120f	3D-2TE -> 3D-3TEN	IL-6	Immune regulatory	-0.574	-1.102	0.047	unchanged	emergent
BC	MDA-231	BN120f	3D-2TE -> 3D-3TEN	IL-8	Chemokine	-0.362	-0.541	0.022	unchanged	emergent
BC	MDA-231	BN120f	3D-2TE -> 3D-3TEN	IL-10	Immune regulatory	1.157	0.69	1.796	induced	emergent
BC	MDA-231	BN120f	3D-2TE -> 3D-3TEN	IL-15	Immune regulatory	-0.075	-0.327	0.183	unchanged	emergent
BC	MDA-231	BN120f	3D-2TE -> 3D-3TEN	IP-10	Chemokine	-2.866	-3.231	-2.408	suppressed	emergent
BC	MDA-231	BN120f	3D-2TE -> 3D-3TEN	LIF	Growth factor	3.33	3.014	3.633	induced	stroma-contributing
BC	MDA-231	BN120f	3D-2TE -> 3D-3TEN	MCP-1	Chemokine	2.725	2.419	3.019	induced	stroma-contributing
BC	MDA-231	BN120f	3D-2TE -> 3D-3TEN	MCP-3	Chemokine	2.795	2.486	3.143	induced	stroma-contributing
BC	MDA-231	BN120f	3D-2TE -> 3D-3TEN	M-CSF	Growth factor	-0.34	-0.671	0.161	unchanged	emergent
BC	MDA-231	BN120f	3D-2TE -> 3D-3TEN	MIF	Immune regulatory	-0.792	-1.208	-0.144	unchanged	emergent
BC	MDA-231	BN120f	3D-2TE -> 3D-3TEN	MIP-1a	Chemokine	2.682	2.403	2.982	induced	stroma-contributing
BC	MDA-231	BN120f	3D-2TE -> 3D-3TEN	MIP-1b	Chemokine	-0.122	-0.351	0.091	unchanged	emergent
BC	MDA-231	BN120f	3D-2TE -> 3D-3TEN	b-NGF	Growth factor	-0.526	-0.835	-0.2	unchanged	emergent

BC	MDA-231	BN120f	3D-2TE -> 3D-3TEN	RANTES	Chemokine	-0.205	-0.639	0.224	unchanged	emergent
BC	MDA-231	BN120f	3D-2TE -> 3D-3TEN	SCGF-b	Growth factor	1.511	1.222	1.816	induced	stroma-contributing
BC	MDA-231	BN120f	3D-2TE -> 3D-3TEN	SDF-1a	Chemokine	-0.161	-0.656	0.413	unchanged	emergent
BC	MDA-231	BN120f	3D-2TE -> 3D-3TEN	TNFa	Immune regulatory	-0.385	-0.62	-0.076	unchanged	emergent
BC	MDA-231	BN120f	3D-2TE -> 3D-3TEN	TNFb	Immune regulatory	-0.802	-1.346	-0.289	unchanged	emergent
BC	MDA-231	BN120f	3D-2TE -> 3D-3TEN	VEGF	Growth factor	-0.269	-0.529	-0.016	unchanged	emergent
BC	MDA-231	BrC4f	3D-2TE -> 3D-3TEC	CTACK	Chemokine	1.262	-0.08	2.241	induced	emergent
BC	MDA-231	BrC4f	3D-2TE -> 3D-3TEC	Eotaxin	Chemokine	0.263	-0.02	0.55	unchanged	emergent
BC	MDA-231	BrC4f	3D-2TE -> 3D-3TEC	b-FGF	Growth factor	-3.04	-3.727	-2.111	suppressed	emergent
BC	MDA-231	BrC4f	3D-2TE -> 3D-3TEC	G-CSF	Growth factor	-0.795	-1.5	0.251	unchanged	emergent
BC	MDA-231	BrC4f	3D-2TE -> 3D-3TEC	GM-CSF	Growth factor	-2.074	-2.562	-1.438	suppressed	emergent
BC	MDA-231	BrC4f	3D-2TE -> 3D-3TEC	GRO-a	Chemokine	-0.703	-1.086	-0.203	unchanged	emergent
BC	MDA-231	BrC4f	3D-2TE -> 3D-3TEC	HGF	Growth factor	3.706	3.32	4.094	induced	stroma-contributing
BC	MDA-231	BrC4f	3D-2TE -> 3D-3TEC	IFN-alpha2	Immune regulatory	0.378	0.168	0.589	unchanged	emergent
BC	MDA-231	BrC4f	3D-2TE -> 3D-3TEC	IFNg	Immune regulatory	-0.573	-0.787	-0.289	unchanged	emergent
BC	MDA-231	BrC4f	3D-2TE -> 3D-3TEC	IL-1a	Immune regulatory	1.011	0.52	1.634	unchanged	emergent
BC	MDA-231	BrC4f	3D-2TE -> 3D-3TEC	IL-1b	Immune regulatory	-0.048	-0.321	0.227	unchanged	emergent
BC	MDA-231	BrC4f	3D-2TE -> 3D-3TEC	IL-1ra	Immune regulatory	0.181	-0.021	0.421	unchanged	emergent
BC	MDA-231	BrC4f	3D-2TE -> 3D-3TEC	IL-2Ralpha	Immune regulatory	0.617	0.159	1.091	unchanged	emergent
BC	MDA-231	BrC4f	3D-2TE -> 3D-3TEC	IL-6	Immune regulatory	0.737	0.253	1.203	unchanged	emergent
BC	MDA-231	BrC4f	3D-2TE -> 3D-3TEC	IL-8	Chemokine	0.295	0.027	0.599	unchanged	emergent
BC	MDA-231	BrC4f	3D-2TE -> 3D-3TEC	IL-10	Immune regulatory	-0.789	-1.337	-0.16	unchanged	emergent
BC	MDA-231	BrC4f	3D-2TE -> 3D-3TEC	IL-15	Immune regulatory	-0.198	-0.555	0.194	unchanged	emergent
BC	MDA-231	BrC4f	3D-2TE -> 3D-3TEC	IP-10	Chemokine	-1.711	-1.966	-1.255	suppressed	emergent
BC	MDA-231	BrC4f	3D-2TE -> 3D-3TEC	LIF	Growth factor	5.467	5.076	5.829	induced	stroma-contributing
BC	MDA-231	BrC4f	3D-2TE -> 3D-3TEC	MCP-1	Chemokine	2.742	2.454	3.023	induced	stroma-contributing
BC	MDA-231	BrC4f	3D-2TE -> 3D-3TEC	MCP-3	Chemokine	6.167	5.923	6.502	induced	stroma-contributing
BC	MDA-231	BrC4f	3D-2TE -> 3D-3TEC	M-CSF	Growth factor	0.031	-0.502	0.579	unchanged	emergent
BC	MDA-231	BrC4f	3D-2TE -> 3D-3TEC	MIF	Immune regulatory	-0.224	-0.674	0.43	unchanged	emergent

https://doi.org/10.3390/xxxxx

BC	MDA-231	BrC4f	3D-2TE -> 3D-3TEC	MIP-1a	Chemokine	4.867	4.586	5.178	induced	emergent
BC	MDA-231	BrC4f	3D-2TE -> 3D-3TEC	MIP-1b	Chemokine	0.114	-0.124	0.348	unchanged	emergent
BC	MDA-231	BrC4f	3D-2TE -> 3D-3TEC	b-NGF	Growth factor	-0.507	-0.79	-0.182	unchanged	emergent
BC	MDA-231	BrC4f	3D-2TE -> 3D-3TEC	RANTES	Chemokine	1.717	-0.008	2.533	induced	emergent
BC	MDA-231	BrC4f	3D-2TE -> 3D-3TEC	SCGF-b	Growth factor	1.536	1.217	1.86	induced	stroma-contributing
BC	MDA-231	BrC4f	3D-2TE -> 3D-3TEC	SDF-1a	Chemokine	0.831	0.339	1.385	unchanged	emergent
BC	MDA-231	BrC4f	3D-2TE -> 3D-3TEC	TNFa	Immune regulatory	0.192	-0.031	0.498	unchanged	emergent
BC	MDA-231	BrC4f	3D-2TE -> 3D-3TEC	TNFb	Immune regulatory	0.365	-0.672	1.252	unchanged	emergent
BC	MDA-231	BrC4f	3D-2TE -> 3D-3TEC	VEGF	Growth factor	-0.35	-0.707	-0.005	unchanged	emergent
BC	MCF7	BN120f	3D-2LE-> 3D-3LEN	CTACK	Chemokine	1.217	0.728	1.703	induced	stroma-contributing
BC	MCF7	BN120f	3D-2LE-> 3D-3LEN	Eotaxin	Chemokine	3.242	2.433	4.182	induced	stroma-contributing
BC	MCF7	BN120f	3D-2LE-> 3D-3LEN	b-FGF	Growth factor	-2.992	-3.255	-2.729	suppressed	emergent
BC	MCF7	BN120f	3D-2LE-> 3D-3LEN	G-CSF	Growth factor	6.34	5.904	6.784	induced	stroma-contributing
BC	MCF7	BN120f	3D-2LE-> 3D-3LEN	GM-CSF	Growth factor	0.14	-0.108	0.388	unchanged	stroma-contributing
BC	MCF7	BN120f	3D-2LE-> 3D-3LEN	GRO-a	Chemokine	2.33	2.137	2.529	induced	stroma-contributing
BC	MCF7	BN120f	3D-2LE-> 3D-3LEN	HGF	Growth factor	4.603	4.206	5.344	induced	stroma-contributing
BC	MCF7	BN120f	3D-2LE-> 3D-3LEN	IFN-alpha2	Immune regulatory	1.202	1.079	1.325	induced	stroma-contributing
BC	MCF7	BN120f	3D-2LE-> 3D-3LEN	IFNg	Immune regulatory	0.274	-0.03	0.579	unchanged	emergent
BC	MCF7	BN120f	3D-2LE-> 3D-3LEN	IL-1a	Immune regulatory	0.458	0.256	0.659	unchanged	stroma-contributing
BC	MCF7	BN120f	3D-2LE-> 3D-3LEN	IL-1b	Immune regulatory	2.565	1.91	3.264	induced	stroma-contributing
BC	MCF7	BN120f	3D-2LE-> 3D-3LEN	IL-1ra	Immune regulatory	0.527	0.208	0.797	unchanged	stroma-contributing
BC	MCF7	BN120f	3D-2LE-> 3D-3LEN	IL-2Ralpha	Immune regulatory	0.401	0.135	0.643	unchanged	stroma-contributing

BC	MCF7	BN120f	3D-2LE-> 3D-3LEN	IL-6	Immune regulatory	4.706	4.501	4.922	induced	stroma-contributing
BC	MCF7	BN120f	3D-2LE-> 3D-3LEN	IL-8	Chemokine	3.63	3.413	3.872	induced	stroma-contributing
BC	MCF7	BN120f	3D-2LE-> 3D-3LEN	IL-10	Immune regulatory	4.119	3.737	4.536	induced	stroma-contributing
BC	MCF7	BN120f	3D-2LE-> 3D-3LEN	IL-15	Immune regulatory	-0.112	-0.335	0.109	unchanged	emergent
BC	MCF7	BN120f	3D-2LE-> 3D-3LEN	IP-10	Chemokine	0.952	0.752	1.142	unchanged	emergent
BC	MCF7	BN120f	3D-2LE-> 3D-3LEN	LIF	Growth factor	5.68	5.39	5.973	induced	stroma-contributing
BC	MCF7	BN120f	3D-2LE-> 3D-3LEN	MCP-1	Chemokine	5.238	5.028	5.464	induced	stroma-contributing
BC	MCF7	BN120f	3D-2LE-> 3D-3LEN	MCP-3	Chemokine	3.551	3.346	3.751	induced	stroma-contributing
BC	MCF7	BN120f	3D-2LE-> 3D-3LEN	M-CSF	Growth factor	0.71	0.52	0.903	unchanged	stroma-contributing
BC	MCF7	BN120f	3D-2LE-> 3D-3LEN	MIF	Immune regulatory	-0.973	-1.343	-0.61	unchanged	emergent
BC	MCF7	BN120f	3D-2LE-> 3D-3LEN	MIP-1a	Chemokine	0.629	0.404	0.843	unchanged	stroma-contributing
BC	MCF7	BN120f	3D-2LE-> 3D-3LEN	MIP-1b	Chemokine	0.936	0.78	1.082	unchanged	stroma-contributing
BC	MCF7	BN120f	3D-2LE-> 3D-3LEN	b-NGF	Growth factor	1.82	1.239	2.715	induced	emergent
BC	MCF7	BN120f	3D-2LE-> 3D-3LEN	RANTES	Chemokine	0.1	-0.093	0.302	unchanged	emergent
BC	MCF7	BN120f	3D-2LE-> 3D-3LEN	SCGF-b	Growth factor	1.494	1.333	1.663	induced	stroma-contributing
BC	MCF7	BN120f	3D-2LE-> 3D-3LEN	SDF-1a	Chemokine	0.039	-0.212	0.321	unchanged	emergent
BC	MCF7	BN120f	3D-2LE-> 3D-3LEN	TNFa	Immune regulatory	0.657	0.09	1.203	unchanged	stroma-contributing
BC	MCF7	BN120f	3D-2LE-> 3D-3LEN	TNFb	Immune regulatory	1.962	1.511	2.482	induced	stroma-contributing
BC	MCF7	BN120f	3D-2LE-> 3D-3LEN	VEGF	Growth factor	-0.252	-0.509	0.002	unchanged	emergent
BC	MCF7	BrC4f	3D-2LE-> 3D-3LEC	CTACK	Chemokine	1.939	1.356	2.495	induced	stroma-contributing
BC	MCF7	BrC4f	3D-2LE-> 3D-3LEC	Eotaxin	Chemokine	3.806	2.959	4.725	induced	stroma-contributing
BC	MCF7	BrC4f	3D-2LE-> 3D-3LEC	b-FGF	Growth factor	-1.191	-1.529	-0.916	suppressed	emergent

https://doi.org/10.3390/xxxxx

BC	MCF7	BrC4f	3D-2LE-> 3D-3LEC	G-CSF	Growth factor	6.074	4.831	6.53	induced	stroma-contributing
BC	MCF7	BrC4f	3D-2LE-> 3D-3LEC	GM-CSF	Growth factor	3.117	1.829	3.614	induced	stroma-contributing
BC	MCF7	BrC4f	3D-2LE-> 3D-3LEC	GRO-a	Chemokine	2.619	2.247	2.949	induced	stroma-contributing
BC	MCF7	BrC4f	3D-2LE-> 3D-3LEC	HGF	Growth factor	5.337	4.89	6.026	induced	stroma-contributing
BC	MCF7	BrC4f	3D-2LE-> 3D-3LEC	IFN-alpha2	Immune regulatory	1.912	1.669	2.094	induced	stroma-contributing
BC	MCF7	BrC4f	3D-2LE-> 3D-3LEC	IFNg	Immune regulatory	0.931	-0.042	1.476	unchanged	emergent
BC	MCF7	BrC4f	3D-2LE-> 3D-3LEC	IL-1a	Immune regulatory	0.928	0.68	1.123	unchanged	stroma-contributing
BC	MCF7	BrC4f	3D-2LE-> 3D-3LEC	IL-1b	Immune regulatory	3.601	2.947	4.265	induced	stroma-contributing
BC	MCF7	BrC4f	3D-2LE-> 3D-3LEC	IL-1ra	Immune regulatory	0.662	0.426	0.886	unchanged	stroma-contributing
BC	MCF7	BrC4f	3D-2LE-> 3D-3LEC	IL-2Ralpha	Immune regulatory	1.073	0.854	1.276	induced	stroma-contributing
BC	MCF7	BrC4f	3D-2LE-> 3D-3LEC	IL-6	Immune regulatory	4.715	4.497	4.939	induced	stroma-contributing
BC	MCF7	BrC4f	3D-2LE-> 3D-3LEC	IL-8	Chemokine	3.766	2.807	4.194	induced	stroma-contributing
BC	MCF7	BrC4f	3D-2LE-> 3D-3LEC	IL-10	Immune regulatory	4.982	4.57	5.422	induced	stroma-contributing
BC	MCF7	BrC4f	3D-2LE-> 3D-3LEC	IL-15	Immune regulatory	-0.02	-0.233	0.191	unchanged	emergent
BC	MCF7	BrC4f	3D-2LE-> 3D-3LEC	IP-10	Chemokine	2.939	2.65	3.154	induced	emergent
BC	MCF7	BrC4f	3D-2LE-> 3D-3LEC	LIF	Growth factor	7.721	7.356	8.061	induced	stroma-contributing
BC	MCF7	BrC4f	3D-2LE-> 3D-3LEC	MCP-1	Chemokine	5.168	4.882	5.466	induced	stroma-contributing
BC	MCF7	BrC4f	3D-2LE-> 3D-3LEC	MCP-3	Chemokine	7.157	6.861	7.437	induced	stroma-contributing
BC	MCF7	BrC4f	3D-2LE-> 3D-3LEC	M-CSF	Growth factor	1.412	0.964	1.725	induced	emergent
BC	MCF7	BrC4f	3D-2LE-> 3D-3LEC	MIF	Immune regulatory	-0.379	-0.878	0.079	unchanged	emergent

BC	MCF7	BrC4f	3D-2LE-> 3D-3LEC	MIP-1a	Chemokine	5.611	5.332	5.878	induced	stroma-contributing
BC	MCF7	BrC4f	3D-2LE-> 3D-3LEC	MIP-1b	Chemokine	1.33	1.038	1.555	induced	stroma-contributing
BC	MCF7	BrC4f	3D-2LE-> 3D-3LEC	b-NGF	Growth factor	2.387	1.807	3.253	induced	emergent
BC	MCF7	BrC4f	3D-2LE-> 3D-3LEC	RANTES	Chemokine	1.1	0.778	1.488	induced	emergent
BC	MCF7	BrC4f	3D-2LE-> 3D-3LEC	SCGF-b	Growth factor	1.662	1.303	1.985	induced	stroma-contributing
BC	MCF7	BrC4f	3D-2LE-> 3D-3LEC	SDF-1a	Chemokine	1.183	0.78	1.502	induced	emergent
BC	MCF7	BrC4f	3D-2LE-> 3D-3LEC	TNFa	Immune regulatory	1.999	1.567	2.376	induced	emergent
BC	MCF7	BrC4f	3D-2LE-> 3D-3LEC	TNFb	Immune regulatory	3.088	2.442	3.68	induced	stroma-contributing
BC	MCF7	BrC4f	3D-2LE-> 3D-3LEC	VEGF	Growth factor	-0.419	-0.944	0.082	unchanged	emergent
BC	SK-BR-3	BN120f	3D-2HE -> 3D-3HEN	CTACK	Chemokine	0.803	0.405	1.228	unchanged	stroma-contributing
BC	SK-BR-3	BN120f	3D-2HE -> 3D-3HEN	Eotaxin	Chemokine	1.864	1.35	2.365	induced	stroma-contributing
BC	SK-BR-3	BN120f	3D-2HE -> 3D-3HEN	b-FGF	Growth factor	-2.87	-3.008	-2.741	suppressed	emergent
BC	SK-BR-3	BN120f	3D-2HE -> 3D-3HEN	G-CSF	Growth factor	1.977	1.711	2.246	induced	stroma-contributing
BC	SK-BR-3	BN120f	3D-2HE -> 3D-3HEN	GM-CSF	Growth factor	0.24	-0.002	0.464	unchanged	stroma-contributing
BC	SK-BR-3	BN120f	3D-2HE -> 3D-3HEN	GRO-a	Chemokine	2.326	2.103	2.55	induced	stroma-contributing
BC	SK-BR-3	BN120f	3D-2HE -> 3D-3HEN	HGF	Growth factor	2.499	2.34	2.681	induced	stroma-contributing
BC	SK-BR-3	BN120f	3D-2HE -> 3D-3HEN	IFN-alpha2	Immune regulatory	1.113	0.677	1.505	induced	stroma-contributing
BC	SK-BR-3	BN120f	3D-2HE -> 3D-3HEN	IFNg	Immune regulatory	2.258	0.943	4.415	induced	stroma-contributing
BC	SK-BR-3	BN120f	3D-2HE -> 3D-3HEN	IL-1a	Immune regulatory	0.545	0.155	0.828	unchanged	stroma-contributing
BC	SK-BR-3	BN120f	3D-2HE -> 3D-3HEN	IL-1b	Immune regulatory	2.102	1.033	4.163	induced	stroma-contributing
BC	SK-BR-3	BN120f	3D-2HE -> 3D-3HEN	IL-1ra	Immune regulatory	0.441	0.173	0.665	unchanged	stroma-contributing

https://doi.org/10.3390/xxxxx

BC	SK-BR-3	BN120f	3D-2HE -> 3D-3HEN	IL-2Ralpha	Immune regulatory	0.356	0.063	0.663	unchanged	stroma-contributing
BC	SK-BR-3	BN120f	3D-2HE -> 3D-3HEN	IL-6	Immune regulatory	5.444	5.195	5.663	induced	stroma-contributing
BC	SK-BR-3	BN120f	3D-2HE -> 3D-3HEN	IL-8	Chemokine	6.62	6.308	6.85	induced	stroma-contributing
BC	SK-BR-3	BN120f	3D-2HE -> 3D-3HEN	IL-10	Immune regulatory	2.208	1.799	2.66	induced	stroma-contributing
BC	SK-BR-3	BN120f	3D-2HE -> 3D-3HEN	IL-15	Immune regulatory	-0.201	-0.41	0.004	unchanged	emergent
BC	SK-BR-3	BN120f	3D-2HE -> 3D-3HEN	IP-10	Chemokine	3.16	2.968	3.37	induced	stroma-contributing
BC	SK-BR-3	BN120f	3D-2HE -> 3D-3HEN	LIF	Growth factor	4.78	4.601	4.961	induced	stroma-contributing
BC	SK-BR-3	BN120f	3D-2HE -> 3D-3HEN	MCP-1	Chemokine	0.154	-0.254	0.475	unchanged	stroma-contributing
BC	SK-BR-3	BN120f	3D-2HE -> 3D-3HEN	MCP-3	Chemokine	2.339	2.21	2.527	induced	stroma-contributing
BC	SK-BR-3	BN120f	3D-2HE -> 3D-3HEN	M-CSF	Growth factor	1.236	1.046	1.46	induced	stroma-contributing
BC	SK-BR-3	BN120f	3D-2HE -> 3D-3HEN	MIF	Immune regulatory	0.483	0.201	0.81	unchanged	stroma-contributing
BC	SK-BR-3	BN120f	3D-2HE -> 3D-3HEN	MIP-1a	Chemokine	5.233	4.997	5.461	induced	stroma-contributing
BC	SK-BR-3	BN120f	3D-2HE -> 3D-3HEN	MIP-1b	Chemokine	1.255	1.045	1.547	induced	stroma-contributing
BC	SK-BR-3	BN120f	3D-2HE -> 3D-3HEN	b-NGF	Growth factor	0.802	0.562	1.038	unchanged	emergent
BC	SK-BR-3	BN120f	3D-2HE -> 3D-3HEN	RANTES	Chemokine	0.348	0.106	0.557	unchanged	emergent
BC	SK-BR-3	BN120f	3D-2HE -> 3D-3HEN	SCGF-b	Growth factor	1.391	1.074	1.685	induced	stroma-contributing
BC	SK-BR-3	BN120f	3D-2HE -> 3D-3HEN	SDF-1a	Chemokine	1.619	1.265	1.93	induced	stroma-contributing
BC	SK-BR-3	BN120f	3D-2HE -> 3D-3HEN	TNFa	Immune regulatory	1.091	0.433	1.684	induced	stroma-contributing
BC	SK-BR-3	BN120f	3D-2HE -> 3D-3HEN	TNFb	Immune regulatory	1.67	0.758	2.101	induced	stroma-contributing
BC	SK-BR-3	BN120f	3D-2HE -> 3D-3HEN	VEGF	Growth factor	-0.041	-0.288	0.206	unchanged	emergent

BC	SK-BR-3	BrC4f	3D-2HE -> 3D-3HEC	CTACK	Chemokine	1.485	0.994	1.94	induced	stroma-contributing
BC	SK-BR-3	BrC4f	3D-2HE -> 3D-3HEC	Eotaxin	Chemokine	2.896	2.45	3.342	induced	stroma-contributing
BC	SK-BR-3	BrC4f	3D-2HE -> 3D-3HEC	b-FGF	Growth factor	-0.271	-0.415	-0.162	unchanged	emergent
BC	SK-BR-3	BrC4f	3D-2HE -> 3D-3HEC	G-CSF	Growth factor	2.842	2.451	3.164	induced	stroma-contributing
BC	SK-BR-3	BrC4f	3D-2HE -> 3D-3HEC	GM-CSF	Growth factor	3.148	2.964	3.332	induced	stroma-contributing
BC	SK-BR-3	BrC4f	3D-2HE -> 3D-3HEC	GRO-a	Chemokine	2.459	2.19	2.714	induced	stroma-contributing
BC	SK-BR-3	BrC4f	3D-2HE -> 3D-3HEC	HGF	Growth factor	5.55	5.377	5.72	induced	stroma-contributing
BC	SK-BR-3	BrC4f	3D-2HE -> 3D-3HEC	IFN-alpha2	Immune regulatory	1.809	1.486	2.116	induced	stroma-contributing
BC	SK-BR-3	BrC4f	3D-2HE -> 3D-3HEC	IFNg	Immune regulatory	2.26	0.993	4.472	induced	emergent
BC	SK-BR-3	BrC4f	3D-2HE -> 3D-3HEC	IL-1a	Immune regulatory	1.124	0.974	1.28	induced	stroma-contributing
BC	SK-BR-3	BrC4f	3D-2HE -> 3D-3HEC	IL-1b	Immune regulatory	3.075	2.124	5.118	induced	stroma-contributing
BC	SK-BR-3	BrC4f	3D-2HE -> 3D-3HEC	IL-1ra	Immune regulatory	0.879	0.642	1.102	unchanged	stroma-contributing
BC	SK-BR-3	BrC4f	3D-2HE -> 3D-3HEC	IL-2Ralpha	Immune regulatory	1.076	0.935	1.201	induced	stroma-contributing
BC	SK-BR-3	BrC4f	3D-2HE -> 3D-3HEC	IL-6	Immune regulatory	5.528	5.409	5.65	induced	stroma-contributing
BC	SK-BR-3	BrC4f	3D-2HE -> 3D-3HEC	IL-8	Chemokine	7.079	6.862	7.259	induced	stroma-contributing
BC	SK-BR-3	BrC4f	3D-2HE -> 3D-3HEC	IL-10	Immune regulatory	3.56	3.114	4.043	induced	stroma-contributing
BC	SK-BR-3	BrC4f	3D-2HE -> 3D-3HEC	IL-15	Immune regulatory	0.092	-0.104	0.294	unchanged	emergent
BC	SK-BR-3	BrC4f	3D-2HE -> 3D-3HEC	IP-10	Chemokine	5.532	5.427	5.63	induced	stroma-contributing
BC	SK-BR-3	BrC4f	3D-2HE -> 3D-3HEC	LIF	Growth factor	8.694	8.383	8.96	induced	stroma-contributing

https://doi.org/10.3390/xxxxx

BC	SK-BR-3	BrC4f	3D-2HE -> 3D-3HEC	MCP-1	Chemokine	2.112	1.946	2.28	induced	stroma-contributing
BC	SK-BR-3	BrC4f	3D-2HE -> 3D-3HEC	MCP-3	Chemokine	6.61	6.486	6.785	induced	stroma-contributing
BC	SK-BR-3	BrC4f	3D-2HE -> 3D-3HEC	M-CSF	Growth factor	1.701	1.368	2.035	induced	stroma-contributing
BC	SK-BR-3	BrC4f	3D-2HE -> 3D-3HEC	MIF	Immune regulatory	1.494	1.301	1.695	induced	emergent
BC	SK-BR-3	BrC4f	3D-2HE -> 3D-3HEC	MIP-1a	Chemokine	5.394	5.221	5.568	induced	stroma-contributing
BC	SK-BR-3	BrC4f	3D-2HE -> 3D-3HEC	MIP-1b	Chemokine	1.484	1.214	1.788	induced	stroma-contributing
BC	SK-BR-3	BrC4f	3D-2HE -> 3D-3HEC	b-NGF	Growth factor	1.34	1.137	1.521	induced	emergent
BC	SK-BR-3	BrC4f	3D-2HE -> 3D-3HEC	RANTES	Chemokine	0.495	0.309	0.665	unchanged	emergent
BC	SK-BR-3	BrC4f	3D-2HE -> 3D-3HEC	SCGF-b	Growth factor	1.68	1.449	1.916	induced	stroma-contributing
BC	SK-BR-3	BrC4f	3D-2HE -> 3D-3HEC	SDF-1a	Chemokine	2.726	2.347	3.092	induced	stroma-contributing
BC	SK-BR-3	BrC4f	3D-2HE -> 3D-3HEC	TNFa	Immune regulatory	1.738	1.415	2.094	induced	stroma-contributing
BC	SK-BR-3	BrC4f	3D-2HE -> 3D-3HEC	TNFb	Immune regulatory	3.019	2.126	3.578	induced	stroma-contributing
BC	SK-BR-3	BrC4f	3D-2HE -> 3D-3HEC	VEGF	Growth factor	0.069	-0.171	0.326	unchanged	emergent
Glioma	U87MG	HMC3	3D-2GA -> 3D-3GAM	CTACK	Chemokine	-0.101	-0.539	0.353	unchanged	emergent
Glioma	U87MG	HMC3	3D-2GA -> 3D-3GAM	Eotaxin	Chemokine	0.472	0.257	0.728	unchanged	emergent
Glioma	U87MG	HMC3	3D-2GA -> 3D-3GAM	b-FGF	Growth factor	-1.276	-1.495	-1.007	suppressed	emergent
Glioma	U87MG	HMC3	3D-2GA -> 3D-3GAM	G-CSF	Growth factor	1.834	1.436	2.255	induced	emergent
Glioma	U87MG	HMC3	3D-2GA -> 3D-3GAM	GM-CSF	Growth factor	1.533	1.408	1.66	induced	stroma-contributing
Glioma	U87MG	HMC3	3D-2GA -> 3D-3GAM	GRO-a	Chemokine	2.861	2.717	3.024	induced	stroma-contributing
Glioma	U87MG	HMC3	3D-2GA -> 3D-3GAM	HGF	Growth factor	-0.185	-0.358	-0.004	unchanged	emergent
Glioma	U87MG	HMC3	3D-2GA -> 3D-3GAM	IFN-alpha2	Immune regulatory	0.133	-0.17	0.428	unchanged	emergent
Glioma	U87MG	HMC3	3D-2GA -> 3D-3GAM	IFNg	Immune regulatory	0.298	0.085	0.554	unchanged	emergent
Glioma	U87MG	HMC3	3D-2GA -> 3D-3GAM	IL-1a	Immune regulatory	0.432	0.11	0.744	unchanged	stroma-contributing

https://doi.org/10.3390/xxxxx

Glioma	U87MG	HMC3	3D-2GA -> 3D-3GAM	IL-1b	Immune regulatory	0.132	0.008	0.253	unchanged	emergent
Glioma	U87MG	HMC3	3D-2GA -> 3D-3GAM	IL-1ra	Immune regulatory	0.883	0.629	1.141	unchanged	emergent
Glioma	U87MG	HMC3	3D-2GA -> 3D-3GAM	IL-2Ralpha	Immune regulatory	0.371	0.163	0.561	unchanged	emergent
Glioma	U87MG	HMC3	3D-2GA -> 3D-3GAM	IL-6	Immune regulatory	0.236	-0.237	0.67	unchanged	stroma-contributing
Glioma	U87MG	HMC3	3D-2GA -> 3D-3GAM	IL-8	Chemokine	0.364	-0.132	0.809	unchanged	stroma-contributing
Glioma	U87MG	HMC3	3D-2GA -> 3D-3GAM	IL-10	Immune regulatory	0.858	0.636	1.102	unchanged	emergent
Glioma	U87MG	HMC3	3D-2GA -> 3D-3GAM	IL-15	Immune regulatory	0.205	0.016	0.394	unchanged	stroma-contributing
Glioma	U87MG	HMC3	3D-2GA -> 3D-3GAM	IP-10	Chemokine	3.199	2.924	3.398	induced	stroma-contributing
Glioma	U87MG	HMC3	3D-2GA -> 3D-3GAM	LIF	Growth factor	0.137	-0.136	0.337	unchanged	emergent
Glioma	U87MG	HMC3	3D-2GA -> 3D-3GAM	MCP-1	Chemokine	-0.778	-0.897	-0.673	unchanged	emergent
Glioma	U87MG	HMC3	3D-2GA -> 3D-3GAM	MCP-3	Chemokine	0.553	0.436	0.689	unchanged	emergent
Glioma	U87MG	HMC3	3D-2GA -> 3D-3GAM	M-CSF	Growth factor	-0.377	-0.457	-0.261	unchanged	stroma-contributing
Glioma	U87MG	HMC3	3D-2GA -> 3D-3GAM	MIF	Immune regulatory	0.616	0.481	0.752	unchanged	stroma-contributing
Glioma	U87MG	HMC3	3D-2GA -> 3D-3GAM	MIP-1a	Chemokine	2.831	2.66	3.012	induced	emergent
Glioma	U87MG	HMC3	3D-2GA -> 3D-3GAM	MIP-1b	Chemokine	1.445	1.29	1.606	induced	stroma-contributing
Glioma	U87MG	HMC3	3D-2GA -> 3D-3GAM	b-NGF	Growth factor	3.753	3.555	3.954	induced	stroma-contributing
Glioma	U87MG	HMC3	3D-2GA -> 3D-3GAM	RANTES	Chemokine	4.716	4.545	4.892	induced	stroma-contributing
Glioma	U87MG	HMC3	3D-2GA -> 3D-3GAM	SCGF-b	Growth factor	0.042	-0.21	0.3	unchanged	emergent
Glioma	U87MG	HMC3	3D-2GA -> 3D-3GAM	SDF-1a	Chemokine	0.061	-0.115	0.241	unchanged	emergent
Glioma	U87MG	HMC3	3D-2GA -> 3D-3GAM	TNFa	Immune regulatory	1.045	0.536	1.415	unchanged	stroma-contributing
Glioma	U87MG	HMC3	3D-2GA -> 3D-3GAM	TNFb	Immune regulatory	0.87	0.413	1.319	unchanged	stroma-contributing
Glioma	U87MG	HMC3	3D-2GA -> 3D-3GAM	VEGF	Growth factor	0.265	-1.098	1.473	unchanged	emergent
Glioma	U87MG	CCF-STTG1	3D-2GM -> 3D-3GAM	CTACK	Chemokine	0.417	0.1	0.665	unchanged	emergent
Glioma	U87MG	CCF-STTG1	3D-2GM -> 3D-3GAM	Eotaxin	Chemokine	0.526	0.358	0.705	unchanged	emergent

https://doi.org/10.3390/xxxxx

Glioma	U87MG	CCF-STTG1	3D-2GM -> 3D-3GAM	b-FGF	Growth factor	-0.891	-1.094	-0.661	unchanged	emergent
Glioma	U87MG	CCF-STTG1	3D-2GM -> 3D-3GAM	G-CSF	Growth factor	1.121	0.82	1.496	induced	emergent
Glioma	U87MG	CCF-STTG1	3D-2GM -> 3D-3GAM	GM-CSF	Growth factor	-0.023	-0.16	0.125	unchanged	emergent
Glioma	U87MG	CCF-STTG1	3D-2GM -> 3D-3GAM	GRO-a	Chemokine	1.702	1.552	1.883	induced	emergent
Glioma	U87MG	CCF-STTG1	3D-2GM -> 3D-3GAM	HGF	Growth factor	1.732	1.551	1.956	induced	stroma-contributing
Glioma	U87MG	CCF-STTG1	3D-2GM -> 3D-3GAM	IFN-alpha2	Immune regulatory	0.368	0.053	0.667	unchanged	emergent
Glioma	U87MG	CCF-STTG1	3D-2GM -> 3D-3GAM	IFNg	Immune regulatory	0.42	0.104	0.69	unchanged	emergent
Glioma	U87MG	CCF-STTG1	3D-2GM -> 3D-3GAM	IL-1a	Immune regulatory	0.462	0.21	0.751	unchanged	emergent
Glioma	U87MG	CCF-STTG1	3D-2GM -> 3D-3GAM	IL-1b	Immune regulatory	0.594	0.469	0.747	unchanged	emergent
Glioma	U87MG	CCF-STTG1	3D-2GM -> 3D-3GAM	IL-1ra	Immune regulatory	-1.021	-1.37	-0.662	suppressed	emergent
Glioma	U87MG	CCF-STTG1	3D-2GM -> 3D-3GAM	IL-2Ralpha	Immune regulatory	0.331	0.126	0.511	unchanged	emergent
Glioma	U87MG	CCF-STTG1	3D-2GM -> 3D-3GAM	IL-6	Immune regulatory	1.066	0.522	1.603	induced	emergent
Glioma	U87MG	CCF-STTG1	3D-2GM -> 3D-3GAM	IL-8	Chemokine	0.045	-0.411	0.48	unchanged	emergent
Glioma	U87MG	CCF-STTG1	3D-2GM -> 3D-3GAM	IL-10	Immune regulatory	0.671	0.389	0.963	unchanged	emergent
Glioma	U87MG	CCF-STTG1	3D-2GM -> 3D-3GAM	IL-15	Immune regulatory	0.193	-0.012	0.393	unchanged	emergent
Glioma	U87MG	CCF-STTG1	3D-2GM -> 3D-3GAM	IP-10	Chemokine	3.055	2.785	3.32	induced	emergent
Glioma	U87MG	CCF-STTG1	3D-2GM -> 3D-3GAM	LIF	Growth factor	1.058	0.712	1.429	induced	emergent
Glioma	U87MG	CCF-STTG1	3D-2GM -> 3D-3GAM	MCP-1	Chemokine	1.894	1.775	2.086	induced	stroma-contributing
Glioma	U87MG	CCF-STTG1	3D-2GM -> 3D-3GAM	MCP-3	Chemokine	0.641	0.447	0.894	unchanged	emergent
Glioma	U87MG	CCF-STTG1	3D-2GM -> 3D-3GAM	M-CSF	Growth factor	-0.598	-0.914	-0.106	unchanged	emergent
Glioma	U87MG	CCF-STTG1	3D-2GM -> 3D-3GAM	MIF	Immune regulatory	0.417	0.165	0.639	unchanged	emergent
Glioma	U87MG	CCF-STTG1	3D-2GM -> 3D-3GAM	MIP-1a	Chemokine	1.868	1.655	2.104	induced	emergent
Glioma	U87MG	CCF-STTG1	3D-2GM -> 3D-3GAM	MIP-1b	Chemokine	0.973	0.825	1.162	unchanged	emergent
Glioma	U87MG	CCF-STTG1	3D-2GM -> 3D-3GAM	b-NGF	Growth factor	1.587	1.379	1.801	induced	emergent
Glioma	U87MG	CCF-STTG1	3D-2GM -> 3D-3GAM	RANTES	Chemokine	1.402	1.306	1.496	induced	emergent
Glioma	U87MG	CCF-STTG1	3D-2GM -> 3D-3GAM	SCGF-b	Growth factor	0.317	0.137	0.493	unchanged	emergent
Glioma	U87MG	CCF-STTG1	3D-2GM -> 3D-3GAM	SDF-1a	Chemokine	2.997	2.79	3.203	induced	stroma-contributing
Glioma	U87MG	CCF-STTG1	3D-2GM -> 3D-3GAM	TNFa	Immune regulatory	1.133	0.704	1.51	induced	emergent
Glioma	U87MG	CCF-STTG1	3D-2GM -> 3D-3GAM	TNFb	Immune regulatory	0.386	-0.023	0.789	unchanged	emergent
Glioma	U87MG	CCF-STTG1	3D-2GM -> 3D-3GAM	VEGF	Growth factor	2.661	1.526	3.447	induced	stroma-contributing

https://doi.org/10.3390/xxxxx
